# Integrating machine learning and geospatial data analysis for comprehensive flood hazard assessment

**DOI:** 10.1007/s11356-024-34286-7

**Published:** 2024-07-20

**Authors:** Chiranjit Singha, Vikas Kumar Rana, Quoc Bao Pham, Duc C. Nguyen, Ewa Łupikasza

**Affiliations:** 1 Department of Agricultural Engineering, Institute of Agriculture, Visva-Bharati (A Central University), Sriniketan, Birbhum, West Bengal 731236 India; 2 Water Resources Engineering and Management Institute, Faculty of Technology & Engineering, The Maharaja Sayajirao University of Baroda, Gujarat, India; 3 Faculty of Natural Sciences, Institute of Earth Sciences, University of Silesia in Katowice, Będzińska Street 60, 41-200 Sosnowiec, Poland; 4 Department of Mechanics and Bridges, Faculty of Civil Engineering, Silesian University of Technology, Akademicka 5, 44-100 Gliwice, Poland

**Keywords:** Flood assessment, Machine learning, Remote sensing, Flood conditioning factors

## Abstract

Flooding is a major natural hazard worldwide, causing catastrophic damage to communities and infrastructure. Due to climate change exacerbating extreme weather events robust flood hazard modeling is crucial to support disaster resilience and adaptation. This study uses multi-sourced geospatial datasets to develop an advanced machine learning framework for flood hazard assessment in the Arambag region of West Bengal, India. The flood inventory was constructed through Sentinel-1 SAR analysis and global flood databases. Fifteen flood conditioning factors related to topography, land cover, soil, rainfall, proximity, and demographics were incorporated. Rigorous training and testing of diverse machine learning models, including RF, AdaBoost, rFerns, XGB, DeepBoost, GBM, SDA, BAM, monmlp, and MARS algorithms, were undertaken for categorical flood hazard mapping. Model optimization was achieved through statistical feature selection techniques. Accuracy metrics and advanced model interpretability methods like SHAP and Boruta were implemented to evaluate predictive performance. According to the area under the receiver operating characteristic curve (AUC), the prediction accuracy of the models performed was around > 80%. RF achieves an AUC of 0.847 at resampling factor 5, indicating strong discriminative performance. AdaBoost also consistently exhibits good discriminative ability, with AUC values of 0.839 at resampling factor 10. Boruta and SHAP analysis indicated precipitation and elevation as factors most significantly contributing to flood hazard assessment in the study area. Most of the machine learning models pointed out southern portions of the study area as highly susceptible areas. On average, from 17.2 to 18.6% of the study area is highly susceptible to flood hazards. In the feature selection analysis, various nature-inspired algorithms identified the selected input parameters for flood hazard assessment, i.e., elevation, precipitation, distance to rivers, TWI, geomorphology, lithology, TRI, slope, soil type, curvature, NDVI, distance to roads, and gMIS. As per the Boruta and SHAP analyses, it was found that elevation, precipitation, and distance to rivers play the most crucial roles in the decision-making process for flood hazard assessment. The results indicated that the majority of the building footprints (15.27%) are at high and very high risk, followed by those at very low risk (43.80%), low risk (24.30%), and moderate risk (16.63%). Similarly, the cropland area affected by flooding in this region is categorized into five risk classes: very high (16.85%), high (17.28%), moderate (16.07%), low (16.51%), and very low (33.29%). However, this interdisciplinary study contributes significantly towards hydraulic and hydrological modeling for flood hazard management.

**Supplementary Information:**

The online version contains supplementary material available at 10.1007/s11356-024-34286-7.

## Introduction

Floods are one of the most frequent and devastating natural hazards, causing substantial loss of lives and damage to infrastructure worldwide. With climate change exacerbating extreme weather events, flooding is projected to increase in frequency and intensity, escalating risk levels across vulnerable regions. This underscores the need for accurate and robust flood hazard modeling to support disaster risk reduction and sustainable development. Flood hazard modeling has witnessed major advancements with the emergence of geospatial technologies and computational capabilities. In particular, machine learning techniques have shown immense potential for flood modeling, given their ability to handle complex nonlinear relationships between flooding and its influencing factors. However, significant research gaps persist, including limited model interpretability, lack of rigorous validation, and inadequate sensitivity analysis regarding input conditioning factors. Accurate mapping of flood hazards is crucial for effective disaster risk reduction and flood management, but conventional flood mapping approaches face limitations in capturing the complexity of flood dynamics. Statistical and analytical techniques often rely on scarce in situ data, overlook interactions between flood causal factors, and struggle to represent nonlinear processes.

Recent studies demonstrate the value of integrating multi-source spatial datasets within GIS-based modeling frameworks to map flood hazards (Rahmati et al. 2016; Armenakis et al. 2017). Diverse factors, including terrain, land use/land cover, soil type, drainage density, rainfall distribution, and proximity to river networks, influence flood susceptibility and need to be considered (Ullah and Zhang 2020; Mousavi et al. 2019). Multicriteria analysis enables weighting and aggregation of these heterogeneous datasets to delineate flood hazard zones (Gazi et al. 2019; Rahmati et al. 2016). GIS-based modeling, leveraging multi-criteria evaluation (MCE) and the analytical hierarchy process (AHP), has demonstrated promising results in regional and national-scale flood hazard mapping for data-scarce regions (Gazi et al. 2019). Remote sensing data provides additional value by offering updated, high-resolution information on land use dynamics and hydrological variables that influence flood exposure (Armenakis et al. 2017). The integration of various spatial datasets within GIS-MCE frameworks holds significant potential for improving the characterization of complex flood hazards and vulnerability. While methods like random forest (Mobley et al. 2021), artificial neural networks (Carreau and Guinot 2021), support vector machines (Youssef et al. 2022), gradient-boosted decision trees (Deroliya et al. 2022), maximum entropy (Maharjan et al. 2024), weighted linear combination (Mahmoody-Vanolya et al. 2021), analytical hierarchy process (Parsian et al. 2021), logistic regression (Pham et al. 2020), frequency ratio (Waqas et al. 2021), weights of evidence (Costache et al. 2022), decision tree (Chen et al. 2020), ordered weighted averaging (Xiao et al. 2017), and technique for order of preference by similarity to ideal solution (TOPSIS) (Rafiei-Sardooi et al. 2021) and neuro-fuzzy logic (Vafakhah et al. 2020) have enabled some predictive flood modeling, there is ample room for improvement and innovation in techniques that integrate spatial big data. However, the choice of factors varies across studies due to differences in data availability, study scale, and modeling approaches. Saikh and Mondal (2023) created a flood susceptibility map for Eastern India using GIS and various machine learning techniques, including artificial neural network (ANN), support vector machine (SVM), random forest (RF), reduced error pruning tree (REPTree), logistic regression (LR), and bagging, based on 200 flood locations and sixteen flood-influencing factors. The results show that all models performed well, with AUROC scores ranging from 0.889 (LR) to 0.926 (Ensemble). The values for very high flood susceptibility zones in the ensemble results ranged between 4.46 and 6.00. Al-Ruzouq et al. (2024) used the eXtreme Deep Factorisation Machine (xDeepFM), deep neural network, support vector machine, and random forest model for flood susceptibility mapping in the UAE, employing Sentinel-1 data and 13 geo-environmental parameters. The dagging model proved to be the most effective, achieving an accuracy of 90.41% for the validation data. Saber et al. (2023) evaluated three machine learning techniques— random forest (RF), LightGBM, and CatBoost—for creating flood susceptibility maps using ten independent factors and 850 flood locations in Vietnam. The AUC-ROC results were 97.9% for CatBoost, and 99.5% for both LightGBM and RF. According to the models' flood susceptibility maps (FSMs), 10–13% of the total area was found to be highly susceptible to flooding. Singha and Swain (2022) conducted a study to map flood inundation in the lower Indo-Gangetic plains, specifically in the Purba Medinipur district of West Bengal, India. Using synchronized C-band Sentinel-1A synthetic aperture radar images with a Google Earth Engine (GEE) cloud, they found that a significant portion of the district (3978.93 km^2^) was inundated during May 2020 due to heavy rainfall and Cyclone Amphan. The flooding affected 35.93% of agricultural land, 5.03% of built-up areas, and 30.85% of the total population. Validation of the flood inundation map using the AUROC method and four machine learning models revealed that the Naïve Bayes model (AUROC = 92.6%) performed better than SVM (AUROC = 89.9%), RF (AUROC = 89.4%), and logistic regression (AUROC = 88.5%).

High-resolution datasets related to topography, land use, soil characteristics, and climate may not be accessible in all study regions, which is a significant constraint. The spatial scale and resolution requirements of the analysis also influence factor selection. Local-scale flood models may require the inclusion of fine details such as microtopography, while regional assessments rely more on generalized terrain attributes. The choice of modeling techniques, including statistical correlations, multi-criteria evaluation, machine learning algorithms, and others, also guides factor selection. Simpler statistical models may incorporate only a few key variables, while more complex techniques can assimilate diverse datasets. However, certain factors are commonly used across studies and models. Topographic variables such as elevation, slope, and curvature are frequently included, as they directly influence the drainage and accumulation of floodwaters. Land use and land cover provide critical information on surface properties and the potential for runoff generation. Soil drainage characteristics are also widely informative for flood models. Rainfall distribution serves as the primary hydrologic driver of flooding, while distance to rivers and streams helps estimate inundation hazards. Population density and road networks offer proxies for socio-economic vulnerability and exposure.

While the selection of factors in flood hazard mapping is context-specific, key factors such as topography, land use, soil, rainfall, proximity, and demography are widely applied in GIS-based techniques. Traditional flood hazard mapping methods face difficulties in accurately representing the complex dynamics of floods because of their slow processing speeds and limited effectiveness, which stem from insufficient data (Aichi et al. 2024; Waseem et al. 2023; Aydin and Sevgi Birincioğlu 2022; Kotecha et al. 2023; Osman and Das 2023; Debnath et al. 2023). Traditional methods require data collection that is often costly, time-consuming, and inaccessible at the local or regional level, particularly in developing countries. Conversely, the integration of Google Earth Engine Cloud with GIS-RS-ML frameworks offers advanced, dynamic tools capable of providing diverse data for risk management, flood zoning, and forecasting. Given that natural disasters are multidimensional phenomena with a  spatial component, GIS-ML is particularly effective for this type of analysis as it can handle large volumes of spatial data used in flood modeling (Prakash et al. 2024; Mehravar et al. 2023; Saravanan et al. 2023). Flood risk management strategies rely heavily on modeling the hydrological, meteorological, and topographic factors of a catchment area to mitigate flood risks in real time. Prioritizing risk analyses and utilizing these innovative frameworks is essential for timely completion. The study focused on the flood-prone area of Arambag in Hooghly district, India, which has experienced devastating floods in the past, resulting in significant economic and human losses. However, the existing literature lacks comprehensive hazard maps and flood risk assessments for this region, which are crucial for effective disaster management. To address this critical gap, our study aimed to use the integration of GEE-based GIS-RS-ML techniques to develop a systematic, data-driven approach for flood hazard assessment. Statistical and analytical techniques often rely on scarce in situ data, overlook interactions between flood conditioning factors, and struggle to represent nonlinear processes between these flood conditioning factors (Yu et al. 2023; Ha et al. 2022). This underscores the need for accurate and robust flood hazard modeling using machine learning models to support disaster risk reduction and sustainable development, especially in vulnerable regions. The lack of high-resolution hazard maps constrains effective disaster management and adaptation in many flood-prone areas. Further research is imperative to determine the selection of appropriate models that can accurately identify and map flood susceptible regions. This can equip communities with actionable insights for adaptation and resilience.

This study aims to address these research gaps by developing an interpretable and validated machine learning framework for flood hazard categorization in the Arambag region of West Bengal, India. Arambag has a history of recurring flood events and remains highly susceptible to flooding due to its geomorphic settings and climatic influences. The study attempts to model the spatial variability in flood hazards through a combination of Sentinel-1 SAR data analysis, multi-sourced spatial database development, implementation of advanced machine learning algorithms, and predictive performance assessment using robust validation metrics. The specific objectives pursued are the following: (i) creation of an inventory of historical and event-based flood extent using Sentinel-1 SAR and Global Flood Database; (ii) assembly of flood conditioning factors encompassing topography, land cover, soil type, precipitation, and anthropogenic variables; (iii) rigorous training and testing of state-of-the-art machine learning models including RF, AdaBoost, rFerns, XGB, DeepBoost, GBM, SDA, BAM, monmlp, and MARS algorithms for flood classification; (iv) comparative assessment of model accuracy using statistical performance metrics; and (v) determining the sensitivity of flood conditioning factors through predictive analysis.

The proposed approach is expected to provide reliable flood hazard categorization for the study area to support risk-informed planning. More broadly, the research would contribute towards advancing machine learning techniques for flood modeling and risk assessment. The following sections present the detailed materials and methods (Sect. 3), results (Sect. 4), and inferences (Sect. 5) from this study.

## Study area

**Fig. 1:**
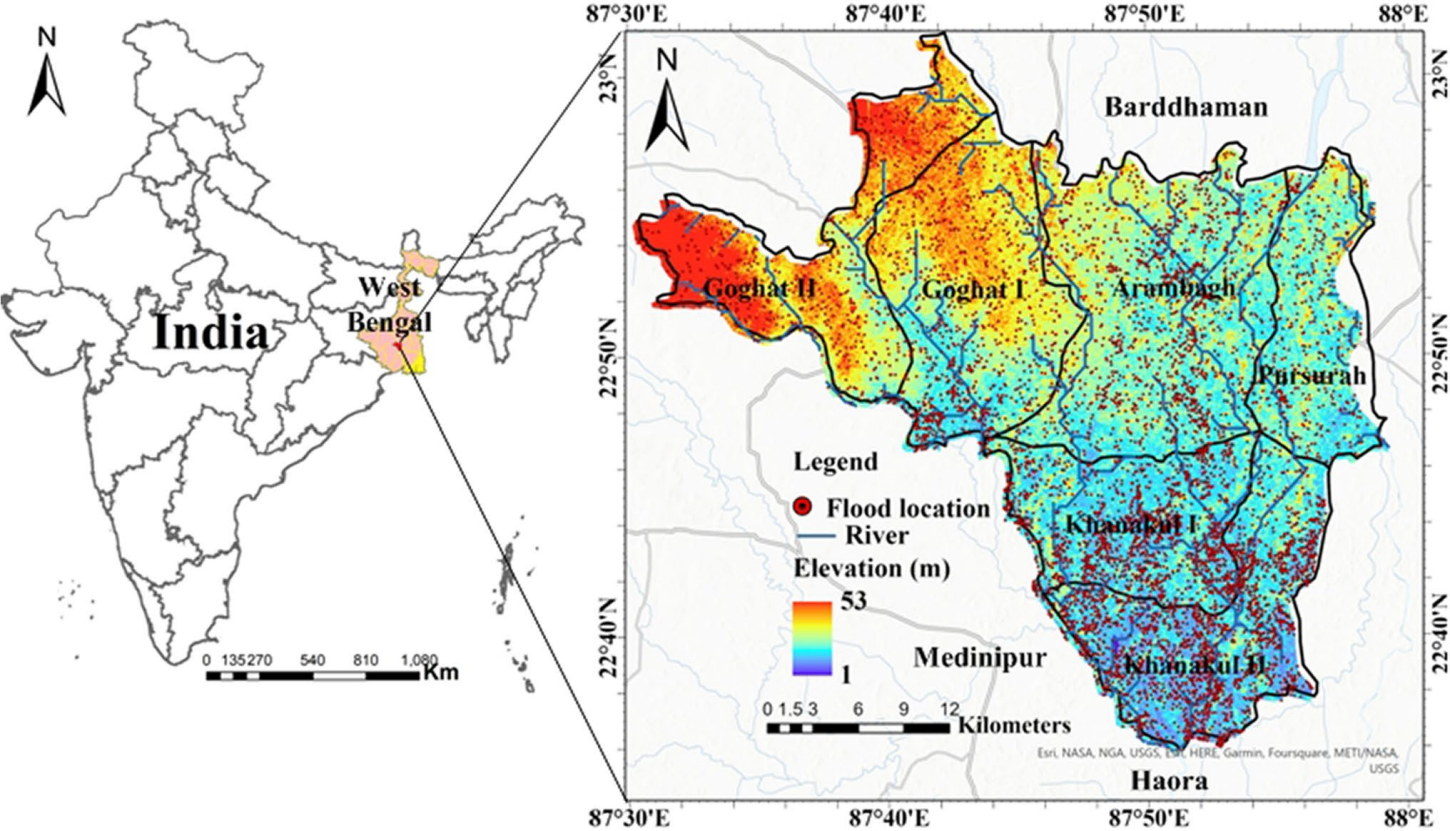
Study area location map

**Fig. 2:**
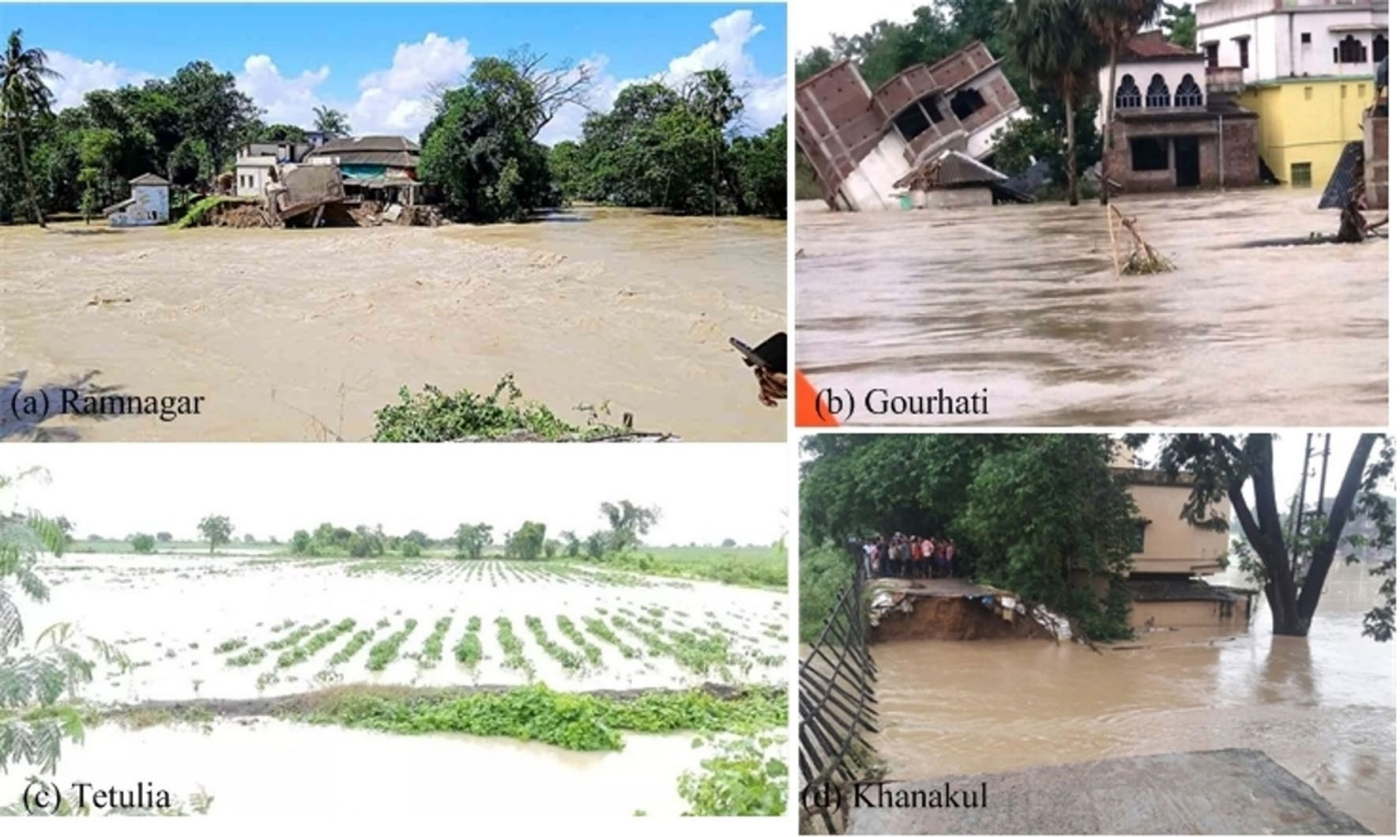
Field photographs related to flood impact of the study region

The study region is situated in the western part of Hooghly district and is considered highly vulnerable to flooding due to its location. The region consists of six adjacent blocks—Goghat I, Goghat II, Arambagh, Pursurah, Khanakul I, and Khanakul II. This region has an area of 1044.44 km^2^ and is located between 22°34′ to 23°1′30′′ N latitude and 87°30′ to 88° E longitude (Fig. 1). It experiences severe downpours, riverbank erosion, and recurrent flooding each year. This region is situated in the interfluve zone between the Dwarkeswar and Damodar Rivers. Also, the Mundeswari, a branch of the Damodar River, flows through this region. Paddy rice is the primary crop, but other crops such as vegetables, potatoes, jute, and orchard products comprise the agricultural economy. Throughout this region, elevation ranges from 1 to 53 m above mean sea level. The climate is tropical with an average annual temperature of around 33 °C and average annual rainfall of around 1300 mm. The western part of the region has red soil, while the rest consists of alluvial plains. The entire region is formed on highly permeable and friable Vindhyan alluvium (Mondal 2016). According to a Disaster Management vulnerability assessment, approximately 40,000 people in the region live in flood-prone zones (District Management Plan, Govt. of West Bengal 2019). Areas including Malayapur, Tirol, Goghat I, Goghat II, Bali, Nakunda, Sheora, Dhadur, Hazirpur, Kunusa, Kumaganj, Mandaria, and Kamarpukur have been severely affected by historical flood events (Government of West Bengal 2015). Figure 2 shows some field photographs collected during flood events in field visits.

## Materials and methods

### Preparation of flood hazard inventory map

The current study utilized the VH polarization of Sentinel-1 GRD’s data (10-m spatial resolution) Two mosaicked images of the data were then produced. The time series of after-flood images showing the flood extent condition of the study area on August 21, 2021, and August 27, 2021, and September 02, 2021, were created with the help of Otsu thresholding approach (Otsu 1979). The pre-flood image acquired on 02nd July 2021 was used for reference. We also used the flood inventory maps for the study region produced by the ESA Copernicus mission (URL: https://scihub.copernicus.eu/). In addition, the long-term historical flood inventory was acquired from the Global Flood Database v1 (2000–2018) in Google Earth engine (GEE) cloud (URL: http://global-flood-database). Some GPS filed survey observed the ground damage for authentication of the ground control points (GCPs) with the field photographs during the flood event on September 02, 2021 (Fig. 2). In this study, S1 SAR, Global Flood Database and filed survey generated 7000 GCPs for creating the 4900 of training (70%) and 2,100 of testing (30%) database of flood hazard inventory maps. The machine learning algorithm’s optimal functioning was determined by randomly assigning points for flood occurrence (value 1) and non-occurrence of flood (value 0) to each of the data points in the study area.

### Methodology

**Fig. 3:**
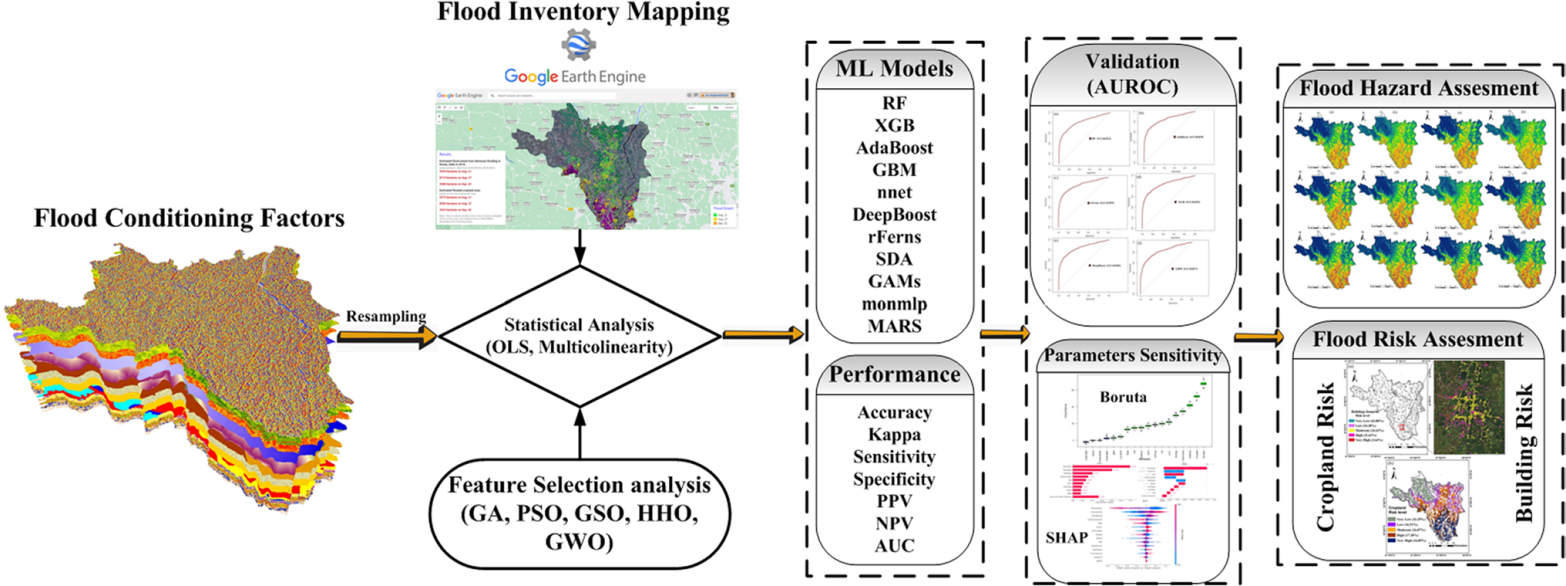
Workflow methodology for Flood hazard mapping

The following workflow of the anticipated steps (Fig. 3) is proceeded into seven parts: (i) GEE platform created the flood inventory based on the S1 SAR and Global Flood Database. (ii) The flood conditioning factors were then used to create a spatial database for flood hazard mapping. (iii) The ordinary least square regression (OLS), multi-collinearity test, and nature-inspired algorithm were utilized to analyze the efficacy and relative selection of the flood conditioning factors. (iv) Several nature-inspired algorithms, including PSO, DFO, GA, GSO, GWO, and HHO, as part of flood hazard modeling approach were employed for feature selection to identify the most significant factors for predicting flood hazards (Table 1). Throughout the feature selection process, the performance of these algorithms was rigorously evaluated based on the objective function. (v) Flood hazard maps were created with RF, AdaBoost, rFerns, XGB, DeepBoost, GBM, SDA, BAM, monmlp, and MARS algorithms. (vi) The ten ML models’ performance was analyzed by different statistical matrices, namely, accuracy, Kappa, sensitivity, specificity, positive predictive values (PPV), negative predictive values (NPV), and area under the receiver operating characteristic curve (AUC). (vii) The Boruta and SHAP analysis applied the model sensitivity and explain the share of each flood hazard conditioning factor.

**Table 1:** Metadata of the utilized datasets for flood hazard mapping

Main factors	Sub-factors		Source	Type
Topographical	Elevation (m)	ASTER DEM, 30 m	URL:https://search.earthdata.nasa.gov/search	Numerical
	Slope (°)			Numerical
	Aspect (°)			Categorical
	Curvature (radians/m)			Numerical
	Topographic Ruggedness Index (TRI)			Numerical
Terrain	Geomorphology	Geological survey of India, 1:25,000	URL: https://www.gsi.gov.in/	Categorical
	Lithology			Categorical
	Land Use Land Cover (LULC)	ESA WorldCover 10 m v200, 10 m, 2021	URL: https://esa-worldcover.org/en	Categorical
Vegetation	Normalized Difference Vegetaion Index (NDVI)	COPERNICUS/S2_SR, 10 m, (2016–2022)	URL: https://scihub.copernicus.eu/	Numerical
Hydro- climatological	Precipitation(mm)	IDAHO_EPSCOR/TERRACLIMATE, 4638.3 m,(1958–2022)	URL: https://www.climatologylab.org/terraclimate.html	Numerical
	Topographic Wetness Index (TWI)	ASTER DEM, 30 m	URL:https://search.earthdata.nasa.gov/search	Numerical
	Distance to river (m)	WWF/HydroSHEDS/v1/FreeFlowingRivers	URL: https://www.hydrosheds.org/	Numerical
Soil	Soil type	NBSSLUP, 1:25,000	URL: https://nbsslup.icar.gov.in/	Categorical
Anthropogenic	Distance to road (m)	OpenStreetMap	URL: https://www.openstreetmap.org	Numerical
	Global Man-made Impervious Surface (gMIS)	Global Man-made Impervious Surface (gMIS) Landsat, v1 (2010)	URL: https://sedac.ciesin.columbia.edu/	Numerical
Flood inventory	SAR data	COPERNICUS/S1_GRD, 10 m	URL: https://sedac.ciesin.columbia.edu/	Numerical
	Flood database	GLOBAL_FLOOD_DB/MODIS_EVENTS/V1, 30 m	URL: http://global-flood-database.cloudtostreet.info/	Numerical

#### Flood conditioning factor

**Fig. 4:**
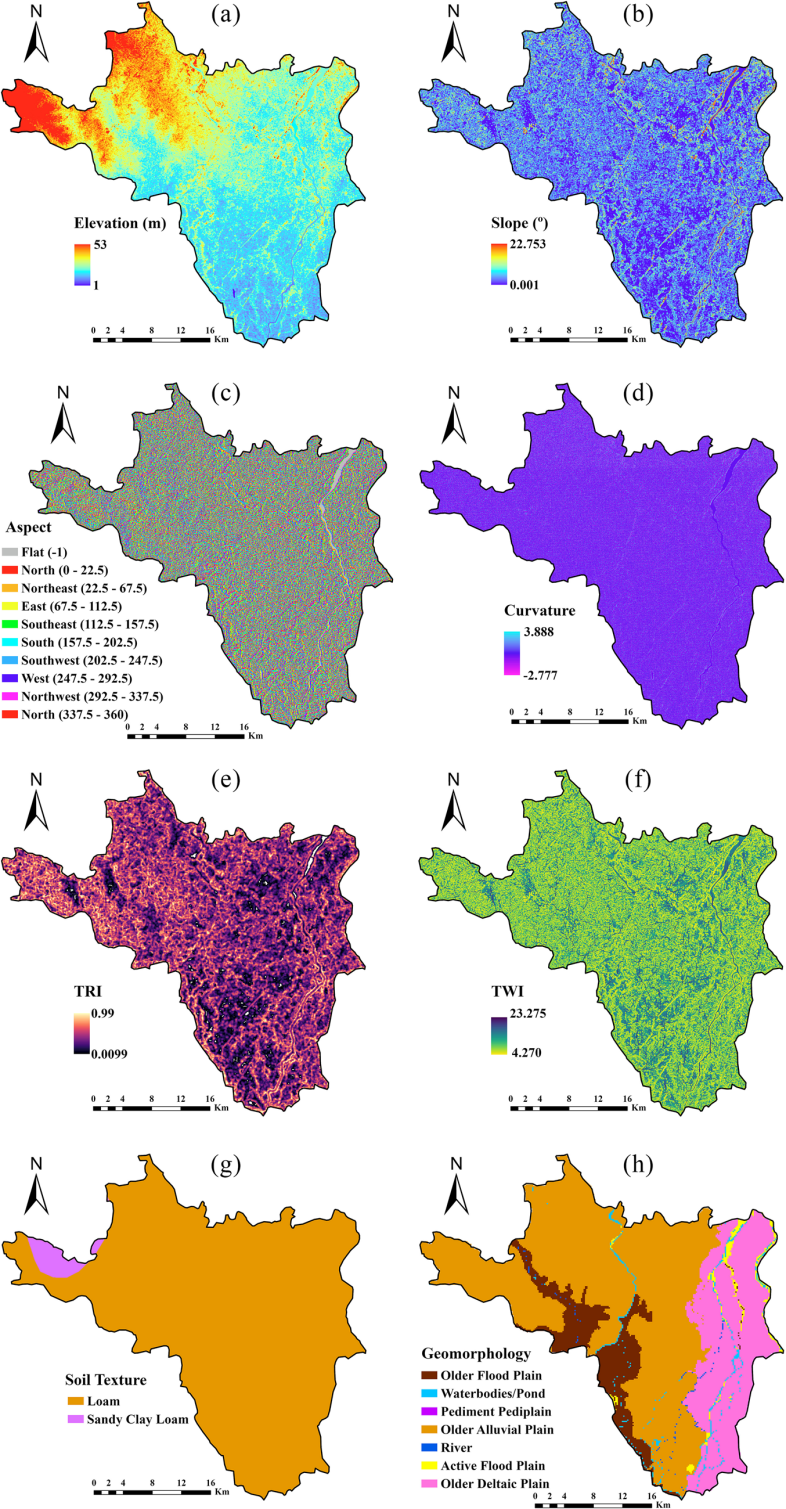

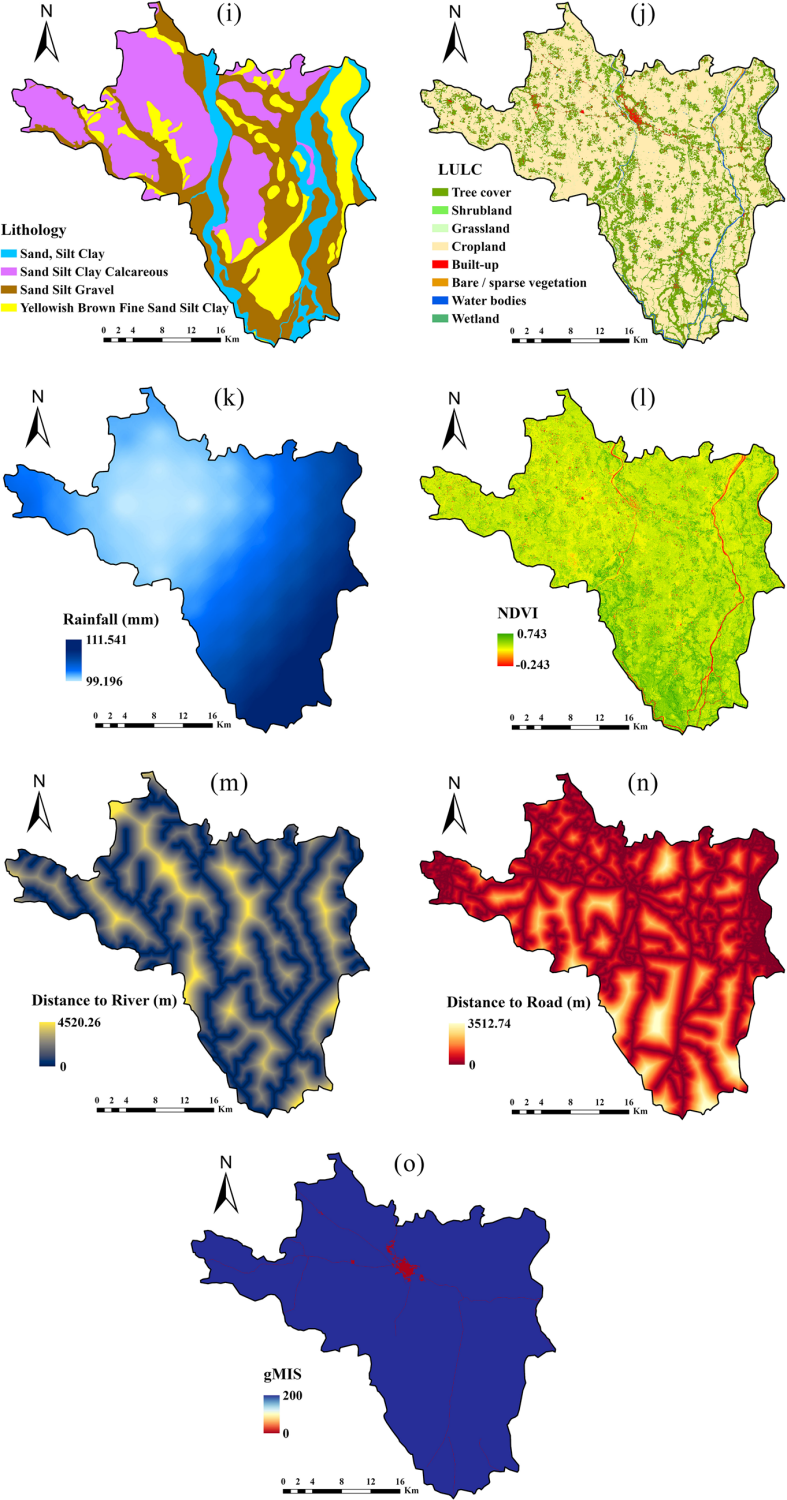
Flood hazard conditioning factors (**a** elevation; **b** slope; **c** aspect; **d** curvature; **e** TRI; **f** TWI; **g** soil texture; **h** geomorphology; **i** lithology; **j** LULC; **k** rainfall; **l** NDVI; **m** distance to river; **n** distance to road; **o** gMIS)

The study used 15 flood-conditioning factors based on previous research to predict the flood hazard map (Swain et al. 2020; Pham et al. 2021; Mehravar et al. 2023), including elevation, aspect, slope, curvature, topographic ruggedness index (TRI), topographic wetness index (TWI), geomorphology, lithology, normalized difference vegetaion index (NDVI), soil texture, land use land cover (LULC), precipitation, distance to river, distance to road, and Global Man-made Impervious Surface (gMIS) (Fig. 4 and Table 1). Some of the flood conditioning factors including elevation, aspect, slope, curvature, topographic TRI, and TWI were generated from the 30 m ASTER DEM. The elevation range in the study region is very low (1 to 53 m), and the area is in the majority covered by alluvial flat plains and cropland. The highest elevation in the study area was located in the northern part (Fig. 4a). The slope angle plays a vital role in controlling the runoff and infiltration on the ground surface (Yariyan et al. 2020). The slope gradient in the study area shifts from north to south, varying between 0.001° and 22.753° (Fig. 4b). An aspect has a reversely proportionate correlation with flood risk, which can be determined by the direction of the water. Since it is linked to physiographic illustrations and soil moisture levels, it can influence the hydrologic outlook (Nachappa et al. 2020). In this study, the aspect map was divided into ten categories, namely flat, north, northeast, east, southeast, south, southwest, west, northwest, and north (Fig. 4c).

The topographic curvature influences surface runoff velocity, flood occurrence, infiltration depth, and soil erosion (Nguyen 2022). The curvature map, created from the DEM, identified three shapes: convex, flat, and concave, with the flat class predominating in the downstream area as depicted in Fig. 4d. Curvature values ranged from − 2.777 to 3.888. TRI inclusion as a flood conditioning factor was also taken into account for local topography variation. For instance, TRI and flood have been in inverse relation (Shafapour Tehrany et al. 2019). ArcGIS generated the TRI map from DEM, with TRI values ranging from 0.009 to 0.99 (Fig. 4e). The TWI is employed to assess the spatial distribution of soil moisture and its influence on erosion. Additionally, it helps in understanding how topography affects surface runoff within a specific geographic area (Tehrany et al. 2019). The average TWI value in the study area was 9.19, with the highest TWI values, around 23.275, occurring downstream and adjacent to the river in the region (Fig. 4f). The TWI was calculated by dividing the area under the influence of the topography by the slope angle (Singha et al. 2022). The TRI and TWI are expressed as (Eqs. 1 & 2).

1 $$TRI=Abs\left(ma{x}^{2}-mi{n}^{2}\right)$$

where max and min denote maximum and minimum elevations, respectively.

2 $$TWI\;=\;Ln\left(\frac{A_s}{tan\beta}\right)$$

where ${A}_{s}$ refers to the specific catchment area, and tan*β* denotes the slope angle at the specific location.

Lithology and geomorphology influence the temporal and spatial variations in sediment generation and the hydrology of the drainage basin (Al-Abadi 2018). Based on the characteristics of the soil and rock permeability and porosity level, lithology determines the runoff and infiltration rate. In addition to indirectly influencing the flood-occurrence process, studies have shown that soil type also has a significant role in controlling runoff risk and soil hydrology on surface to sub surface (Costache et al. 2020). The study region primarily comprises two soil textural classes: loam and sandy clay loam. Loam is the dominant soil texture type across the entire study area (Fig. 4g). The vector layer containing lithology and geomorphology information (scale 1:25,000) was obtained from the Geological Survey of India.

In this study, geomorphology is organized into seven categories: older flood plain, water bodies/ponds, pediment pediplain, older alluvial plain, river, active flood plain, and older deltaic plain (Fig. 4h). The older alluvial plain comprises the largest proportion of the geomorphological units in the study area, accounting for approximately 60%. Regarding lithological classification (Fig. 4i), various types have been identified, including sand, silt, clay, sand with calcareous deposits, sand with gravel, and fine sand with silt and clay, appearing yellowish-brown. The land use map of 2021 was produced using the ESA WorldCover 10 m v200 data in the GEE platform. The study area encompasses around eight land use and land cover (LULC) classes (Fig. 4j), comprising tree cover, shrubland, grassland, cropland, built-up areas, sparse vegetation with little or no vegetation, water bodies, and wetlands. Cropland is the predominant land cover type, accounting for approximately 70% of the area, particularly across the entire study region.

One of the most influential flood conditioning factors is rainfall, which can trigger flooding intensity (Swain et al. 2020). An increase in rainfall intensity can also increase flood intensity. The map of average annual precipitation totals (1958–2022) in the studied region was created based on TerraClimate data using IDW interpolation in ArcGIS v.10.7. Based on the rainfall map, the average minimum rainfall is 99.196 mm in the upstream sections and 111.541 mm in the downstream areas (Fig. 4k). The study area’s annual average rainfall is recorded at 103 mm. Generally, the infiltration rate and runoff speed factor are determined by various criteria such as the NDVI, and land cover (Duong Thi et al. 2020). The Sentinel 2 based mean NDVI map (2016–2022) was created in the GEE platform (Eq. 3). The NDVI ranges between − 2.43 and 0.743, with a mean NDVI value of 0.43 observed across the study area (Fig. 4l).

3 $$\text{NDVI}=\frac{(\text{NIR}-\text{R})}{(\text{NIR}+\text{R})}$$

where *R* denotes the Red band, band 4, and NIR denotes the near-infrared band, band 8.

Also, since the areas near rivers are more prone to flooding, the distance from them is regarded as a vital factor for flood occurrences. Other anthropogenic factors such as distance to road and gMIS were also considered with flood extent due to their impervious level (Kalantari et al. 2014). Then, the Euclidean distance tool was applied to generate the raster map of the distance to river and road factor in the ArcGIS environment. Frequently, flooding initiates from the river or canal riverbed and extends into the surrounding areas. Regions adjacent to water bodies, such as rivers, pose a high risk of flooding, with this risk diminishing as distance from the water source increases.

The distance to the river map extends from 0 to 4520.26 m (Fig. 4m). The range of distances on the road map falls between 0 and 3512.74 m (Fig. 4n). The gMIS maps showed values ranging from 0 to 255, with a value of 0 indicating a high concentration of Global Man-made Impervious Surface (gMIS), notably observed in the town of Arambag in the northern part of the region (Fig. 4o).

#### Ordinary least square (OLS) and multicollinearity test

In this study, OLS method with the linear dependence manner was used to explain the significance of flood occurrence factors. In addition, the multi-collinearity test assessed the intrastate relation among the flood condition factors. The extreme correlation input factors were adjusted in the flood hazard modeling process. The authentication of the Multicollinearity test was applied using the tolerance ($TOL$) (> 0.1) and variance inflation factor ($VIF$) score (< 10) among the independent factors (Singha et al. 2023). TOL and VIF values were calculated using Eq. 4 (Eq. 4). Furthermore, all 15 flood conditioning factors were utilized in the flood hazard modeling process.

4 $$\begin{array}{c}TOL=1-R_j^2\\VIF=\frac1{TOL}\end{array}$$

where the value of ${R}_{j}^{2}$ denotes the value of regression j in different flood condition factors of the dataset.

#### Nature-inspired feature selection techniques

In order to assess the effectiveness and relative significance of flood conditioning factors, it is crucial to recognize the conditioning factor as a pivotal preliminary step preceding the application of model training. The feature selection process tested the model performance or efficiency with their error correction. To achieve that, the previous research used nature-inspired algorithms to appraise and impact the input of flood conditioning factors in flood occurrences (Mehravar et al. 2023; Razavi-Termeh et al. 2023). This algorithm helps to identify the most effective factors for flood hazard modelling. In this study, different nature-inspired algorithms, namely Genetic Algorithm (GA), Particle Swarm Optimization (PSO), Gravitational Search Optimization (GSO), Harris Hawk Optimization (HHO), and Grey Wolf Optimization (GWO), were employed using the *zoofs* python library with light gbm-based optimization technique. Table 2 summarizes the best hyperparameters optimization with several nature-inspired algorithms, including PSO, DFO, GA, GSO, GWO, and HHO, as part of the flood hazard modeling approach were employed for feature selection to identify the most significant factors for predicting flood hazards. Throughout the feature selection process, the performance of these algorithms was rigorously evaluated based on the objective function. The most effective objective functions are utilized in flood hazard assessment, with GSO achieving a score of 0.39013, followed by GA (0.307152), HHO (0.307152), GWO 382 (0.307152), and PSO (0.30708) respectively.

**Table 2:** Best hyper-parameters optimization of nature-inspired algorithms for flood hazard assessment

Algorithm name	Tuning parameters
PSO	n_iteration = 20, Total Bins 1752, pavg = 0.498174, initscore = -0.007303, population_size = 20, Finished iteration #19 with objective value 0.30708779035092854. Current best value is 0.30708779035092854
GSO	n_iteration = 50, population_size = 50, g0 = 100, eps = 0.5, minimize = True, Total Bins 520, Number of data points in the train set: 6847, number of used features: 3, pavg = 0.498174, initscore = -0.007303, Start training from score -0.007303,Finished iteration #9 with objective value 0.39013012872392006. Current best value is 0.3251658438571509
GA	n_iteration = 20, population_size = 20,selective_pressure = 2,elitism = 4, mutation_rate = 0.1,minimize = True, -05, Total Bins 1873, Number of data points in the train set: 6847, number of used features: 13, pavg = 0.498174, initscore = -0.007303,Finished iteration #19 with objective value 0.30715234360321764. Current best value is 0.30715234360321764
HHO	n_iteration = 20, population_size = 20,minimize = True, Finished iteration #19 with objective value 0.30715234360321764. Current best value is 0.30708779035092854
GWO	n_iteration = 20,population_size = 20,minimize = True, Total Bins 2095, Number of data points in the train set: 6847, number of used features: 14, pavg = 0.498174, initscore = -0.007303,Start training from score -0.007303 Finished iteration #19 with objective value 0.30715234360321764. Current best value is 0.30715234360321764

#### Application of machine learning model

The study used binary values to identify locations with and without flood conditions. Machine learning models were trained with an adaptive cross-validation procedure, employing a two-resampling approach with settings of number = 5, repeat = 5, and number = 10, repeat = 10.

The RF algorithm is a supervised learning framework used for classification and regression (Breiman 2001). It consists of multiple decision trees, and its key tuning components are the number of trees, maximum depth, and number of splits. The XGB algorithm addresses overfitting through feature-random and bagging-boosting ensemble techniques (Chen and Guestrin 2016). It uses an additive function to forecast the target variable, modeled as a sum of K discrete CARTs (Classification and Regression Trees). The predicted score (yi) is the sum of the values from each tree applied to the input sample. Adaptive boosting, proposed by Schapire and Freund in 1997, combines decision trees with boosting-based weak classifiers (Zahid et al. 2020). This method creates a Boolean classifier for flood and no-flood conditions and iteratively reweights misclassified samples until a threshold is met, enhancing the classifiers’ performance. Friedman’s Gradient Boosting Machine (GBM), a supervised learning algorithm, uses various loss functions to handle outliers and minimizes the loss function to improve robustness. AdaBoost focuses on reweighting training instances based on difficulty and aims to refine hypotheses continually. The training process involves evaluating the consistency of the VC interval or the Rademacher component (Cortes 2014). The random ferns algorithm explained through a Bayesian derivation, is interpreted as a type of decision tree ensemble (Kursa 2014). The rFerns package highlights two main features of random forests: inner error approximation and feature importance. It effectively handles both categorical and continuous data, maintaining the stochastic nature of random ferns similar to the Extra-trees algorithm (Geurts et al. 2006). SDA is suitable for high-dimensional diagonal and linear discriminant analysis with variable selection (Zuber and Strimmer 2009). It employs a classifier trained using the James–Stein-type shrinkage estimation method and a ranking procedure (CAT scores), useful for classification studies involving correlated predictors. GAMs model relationships using a set of random functions, eliminating the need for a simple weighted sum (Marx and Eilers 1998). The model sums multiple “splines,” providing flexibility and some explanatory power of linear regression. The monmlp is used for applications requiring a monotonic multi-dimensional model (Lang 2005). It ensures weight constraints in multi-layer perceptron networks and maintains monotonicity regardless of training quality. It is applicable in flood hazard mapping for model predictive control. MARS is a non-parametric method combining linear regression, binary partitioning, and splines to create local models (Friedman 1991; Felicisimo et al. 2013).

#### Validation method

Several statistical indices, namely accuracy, Kappa coefficient, sensitivity, specificity, PPV, NPV, and AUC (Eqs. 5, 6, 7, 8, 9), were employed to estimate the efficacy of the flood hazard models.

5 $$\text{Accuracy }=\frac{\text{TP}+\text{TN}}{\text{TP}+\text{TN}+\text{FP}+\text{FN}}$$

6 $$\text{Kappa coefficient }=\frac{{P}_{\text{obs }}-{P}_{\text{exp }}}{1-{P}_{\text{exp}}}$$

7 $$\text{Sensitivity }=\frac{\text{TP}}{\text{TP}+\text{FN}}$$

8 $$\text{Specificity }=\frac{\text{TN}}{\text{TN}+\text{FP}}$$

9 $$PPV=\frac{\text{TP}}{\text{TP}+\text{FP}}$$

$$NPV=\frac{\text{TN}}{\text{TN}+\text{FN}}$$

where TP is true positive, TN is true negative, FP is false positive, and FN is false negative.

Better model presentation is explained by higher accuracy, kappa, and AUC values (Habibi et al. 2023).

#### Parameter sensitivity analysis

This research estimates the parameter sensitivity of the flood condition factors through the Boruta wrapper and SHapley Additive exPlanations (SHAP) algorithm. The input factors related to flood conditions are used to generate SHAP scores to analyze the sensitivity of parameters to flood hazard impact. Boruta and SHAP analysis was carried out by the benchmark RF model in the Google Colab code editor (Kursa and Rudnicki 2010; Lundberg and Lee 2017). The Boruta and SHAP method can be utilized with various kinds of models for flood hazard prediction, including neural networks, linear models, and support vector machine models, respectively (Seleem et al. 2022; Aydin and Iban 2022; Habibi et al. 2023). Generally, SHAP shows significant features and determines if they positively or negatively influence the predicted values. On the other hand, the Boruta algorithm employs the z-score as a criterion to determine if a feature is essential or superfluous (Eq. 10).

10 $$z-\text{ score }=(i-x)/s$$

where *i* denotes the importance value of the predictive sample, *x* is the mean importance value of the conforming shadow sample, and *s* denotes the standard deviation of the importance value of the predictive sample.

## Results and discussion

### OLS and multicollinearity analysis

Anaconda Python Jupyter Notebook version 3.26 was utilized to perform OLS and multicollinearity analyses. The results of this comprehensive analysis are presented in Table 2. According to the OLS analysis, the flood conditioning factors with the highest significance (*p* < 0.001) include geomorphology, elevation, lithology, TWI, precipitation, slope, soil type, curvature, NDVI, land use and land cover (LULC), and distance to the road. The OLS model demonstrates statistical significance, as indicated by the high F-statistic (784.8). However, the presence of autocorrelation in the residuals (Durbin-Watson = 0.421) raises concerns about the reliability of the model’s estimates. Moreover, despite the model’s overall significance, a significant portion of the variance in the dependent variable remains unexplained by the Flood conditioning factors. The *R*-squared value of 0.633 suggests that approximately 63.3% of the variance in the flood probability is accounted for by the flood conditioning factors in the model. The OLS also revealed significant relationships between flood conditioning factors and flood probability in the study area. Curvature, with a *β* of 0.1014 (std error of 0.02) and a *p*-value less than 0.001, exhibited a positive association, indicating that areas with more undulating terrain tend to have a higher flood probability. Similarly, the NDVI displayed a positive *β* of 0.2643 (*p*-value = 0.001), suggesting that regions with higher vegetation cover are more susceptible to flooding in the study area. Notably, precipitation emerged as the most influential factor, with a substantial positive *β* of 1.0089 and a *p*-value less than 0.001. This strong positive relationship implies that as precipitation levels increase, the likelihood of flooding in the study area increases significantly. The variables TRI (*β* =  − 0.0771; *p* = 0.146), aspect (*β* =  − 0.0036; *p* = 0.094), gMIS (*β* =  − 0.0011; *p* = 0.03), and DistRiver (*β* =  − 0.000006; *p* = 0.358) do not reach levels of statistical significance as *p* > 0.001 suggesting non-significant effect on the flood probability. Concurrently, the multicollinearity analysis was conducted to assess the associations between these elements. The analysis revealed a maximum VIF (variance inflation factor) value of 4.16 and the minimum TOL (tolerance) value of 0.24, both of which are detailed in Table 3. Given that no variable exhibits a VIF value exceeding 10 and that the TOL values remain below 0.1, it can be confidently stated that the flood hazard mapping method remains unaffected by issues related to collinearity. The combined results of the OLS and multicollinearity analyses confirm that the selected flood conditioning factors are statistically significant predictors, and their interaction does not pose problems in terms of collinearity. This robust set of factors is further employed in subsequent machine learning modeling for flood hazard mapping.

**Table 3:** OLS and multicollinearity test

Factors	*β*	std err	*t*	*P* >|*t*|	Tolerance	VIF
Geomorphology	− 0.0262	0.003	− 8.927	0***	0.51	1.98
Elevation	− 0.0348	0.001	− 28.727	0***	0.41	2.46
Lithology	0.0354	0.006	5.959	0***	0.83	1.21
TRI	− 0.0771	0.053	− 1.455	0.146	0.64	1.56
TWI	− 0.0161	0.003	− 6.263	0***	0.58	1.73
Precipitation	1.0089	0.115	8.806	0***	0.24	4.16
Slope	− 0.02	0.005	− 4.248	0***	0.78	1.27
SoilType	0.0733	0.014	5.324	0***	0.40	2.49
Curvature	0.1014	0.02	4.967	0***	0.85	1.18
NDVI	0.2643	0.076	3.475	0.001***	0.68	1.47
LULC	0.0346	0.006	5.881	0***	0.63	1.58
Aspect	− 0.0036	0.002	− 1.677	0.094	0.99	1.01
DistRoad	0.0000863	0.00000903	9.56	0***	0.82	1.22
DistRiver	− 0.000006835	0.00000743	− 0.92	0.358	0.95	1.05
gMIS	− 0.0011	0	− 2.173	0.03*	0.98	1.02
	F-statistic = 784.8	Durbin-Watson = 0.421	Prob > chi2 = 0.001		*R*-squared = 0.633	

*β*, coefficient; *t*, t test; *std err*, standard error; robust standard errors ~ *p < 0.05, **p < 0.01, and, ***p < 0.001; *F*, statistical; *R*^*2*^, linear regression

### Implementation of nature-inspired algorithm

The outcomes of this evaluation clearly indicated that GSO (0.39013) emerged as the top-performing algorithm with the best objective function, surpassing GA (0.307152), HH (0.307152), GWO (0.307152), and PSO (0.30708), which closely followed suit. This performance comparison is visually represented in Supplementary Figs. 1a–e. The analysis revealed that the critical factors for flood hazard modeling, identified by these nature-inspired algorithms, include that geomorphology, elevation, lithology, TRI, TWI, precipitation, slope, soil type, curvature, NDVI, distance to road, distance to river, and gMIS. These pivotal factors were meticulously selected through feature selection techniques, underscoring their pivotal role in precisely modeling flood hazards within our study (Supplementary Table 1). Importantly, the alignment of these top factors with the results of statistical analyses further underscores the robustness of the approach.

### Machine model application

**Fig. 5:**
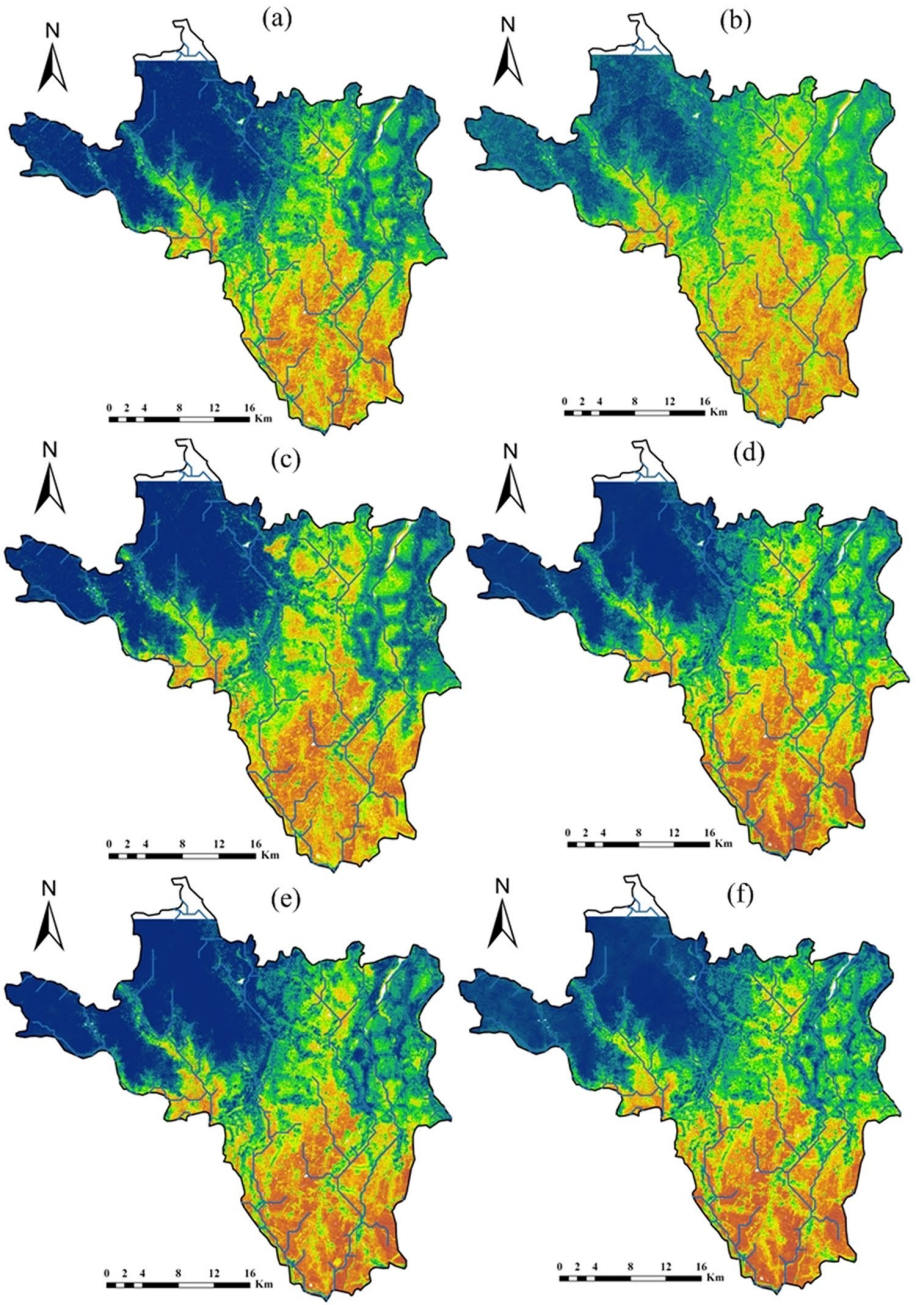

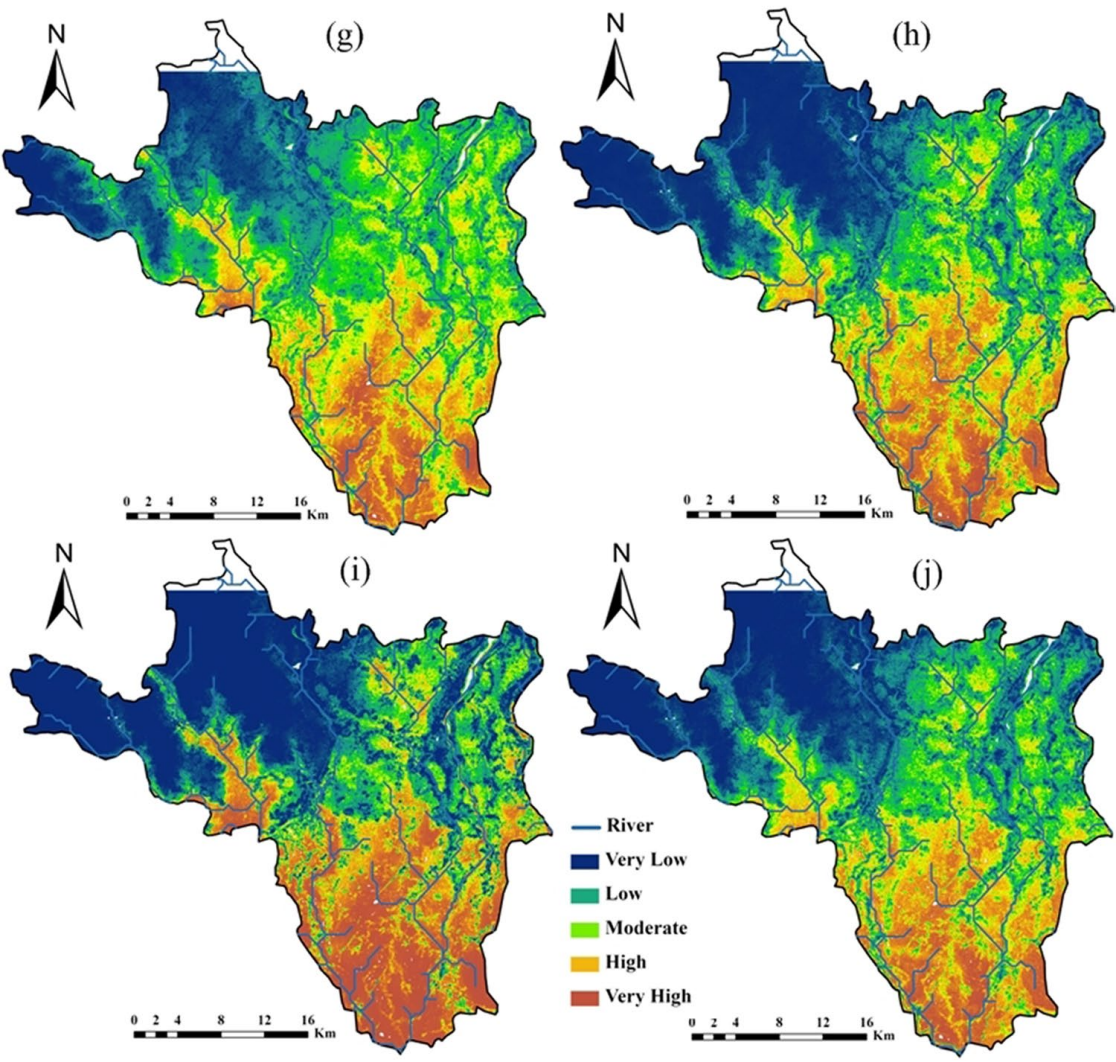
Flood hazard prediction maps with 5 resampling ML technique (**a** RF; **b** AdaBoost; **c** rFerns; **d** XGB; **e** DeepBoost; **f** GBM; **g** SDA; **h** BAM; **i** monmlp; **j** MARS)

**Fig. 6:**
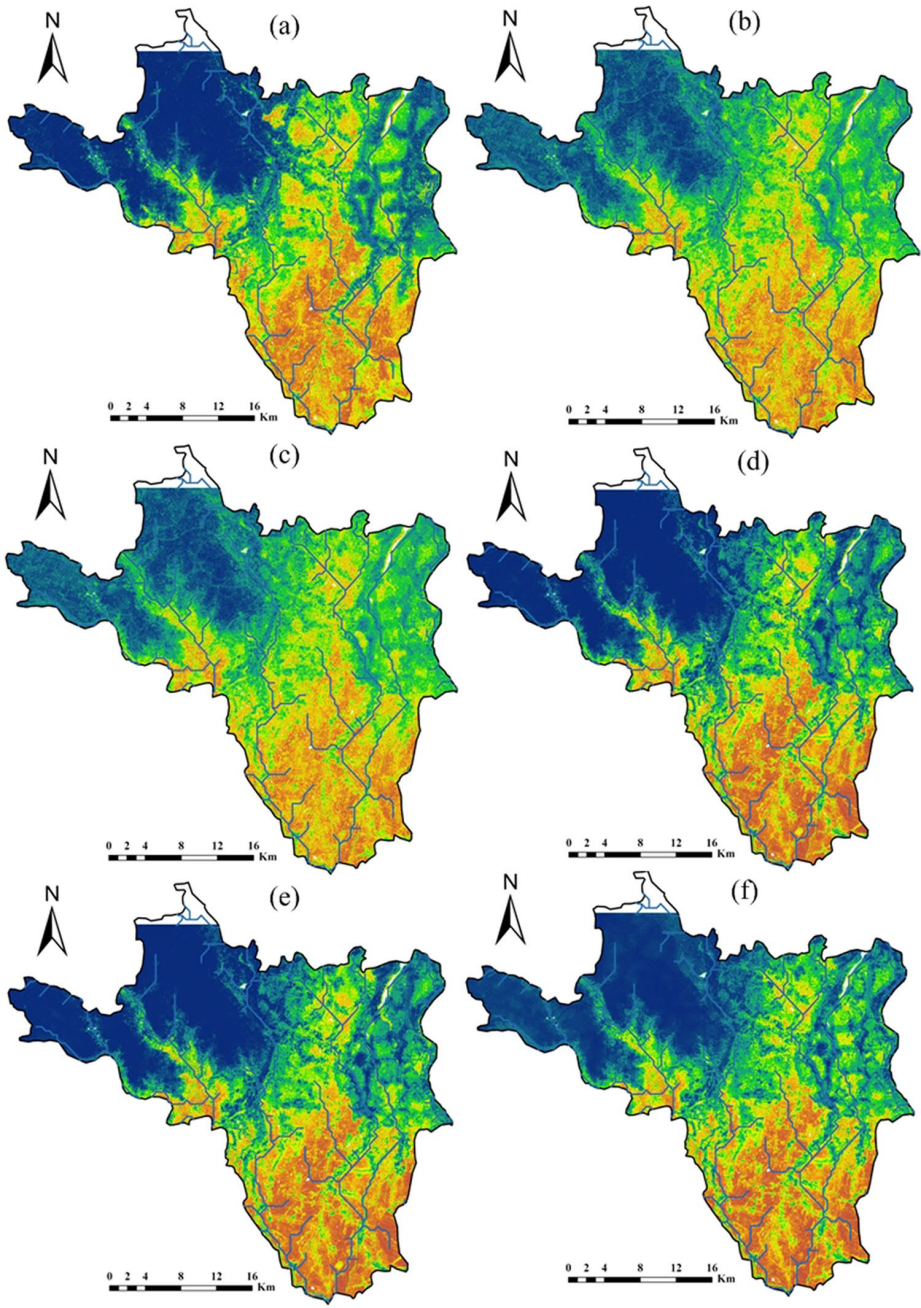

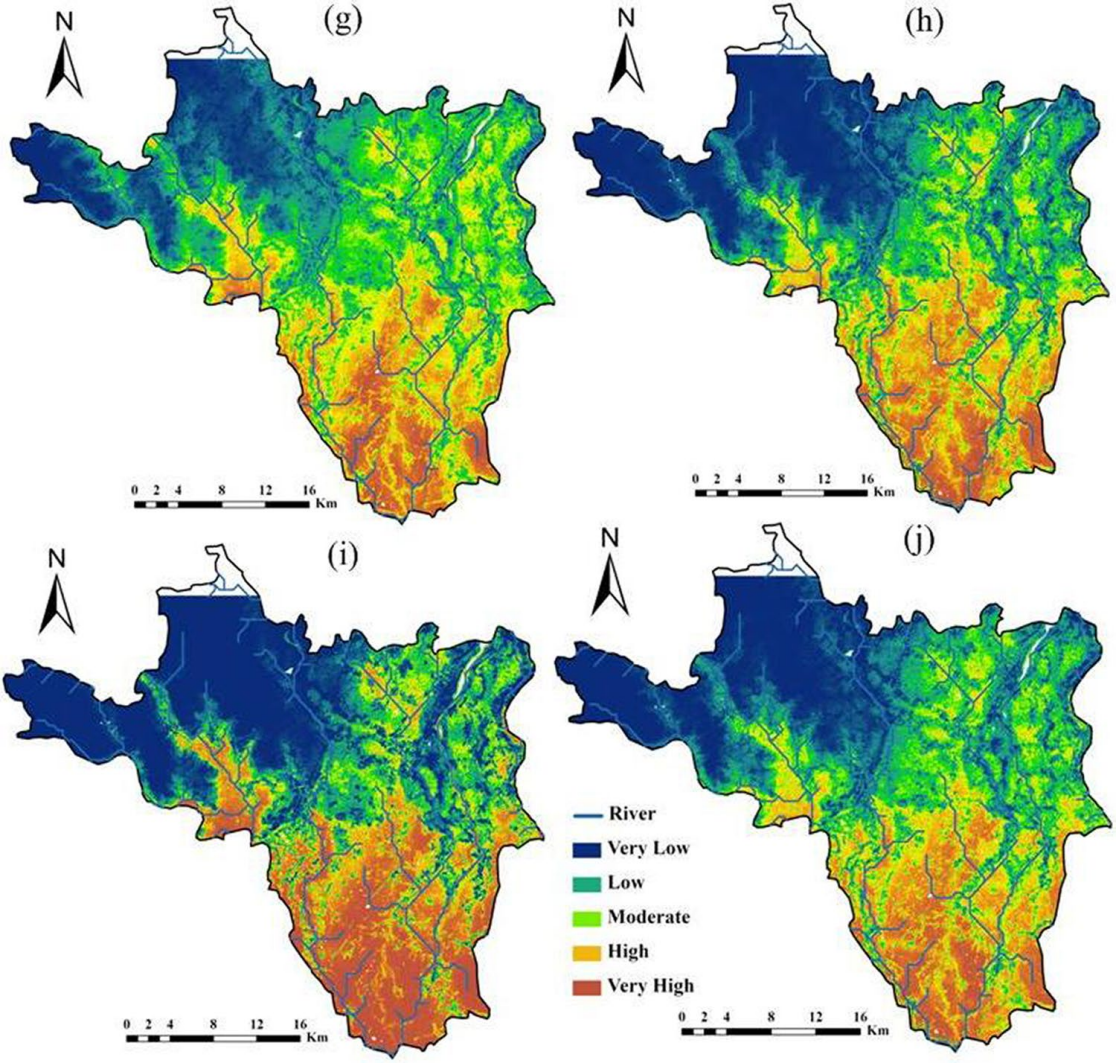
Flood hazard prediction maps with 10 resampling ML (**a** RF; **b** AdaBoost; **c** rFerns; **d** XGB; **e** DeepBoost; **f** GBM; **g** SDA; **h** BAM; **i** monmlp; **j** MARS)

In this section, a comparative performance analysis of ML models in estimating the areal coverage of flood zone categories is presented. The natural break technique used in ArcGIS 10.7 categorized flood hazard zones into five categories: very low, low, moderate, high, and very high, as illustrated in Figs. 5 and 6. The darker blue color in the upper part of the area indicates that the area with very low flood hazard zone, while a darker brown in the southeastern most part of the area represented very high flood hazard zone. The analysis of areal coverage in percentage across various flood hazard categories highlighted the consistency of models in predicting flood hazard zones; this consistency is assessed based on the standard deviation values for each model and flood hazard category for resampling factors 5 and 10, with lower standard deviation values indicating more consistent predictions and higher values suggesting greater variability in model performance.

In Table 5, in the category of “very low” areal coverage percentage, the model rFerns stands out with the highest value of 43.32%, while SDA achieves the lowest at 24.19%. This indicates that rFerns predicts a much larger portion of the area to fall into the “very low” category compared to other models. For the “low” category, AdaBoost records the highest coverage at 21.78%, slightly higher than the next highest, SDA at 21.05%. In contrast, rFerns exhibits the lowest value, covering only 8.29% of the area in this category, indicating a much smaller estimation of “low” risk areas compared to other models. When examining the “moderate” category, SDA leads with 22.35%, suggesting it predicts a higher moderate risk area than others. The lowest value is again recorded by rFerns at 6.74%, demonstrating its tendency to underpredict moderate risk areas relative to its peers. In the “high” category, AdaBoost achieves the highest areal coverage percentage at 19.16%, closely followed by RF at 18.46% and DeepBoost at 18.45%. Monmlp, however, predicts the least area in this category with only 11.74%, indicating a more conservative estimation of high-risk areas. For the “very high” category, rFerns predicts the largest area at 24.84%, suggesting a significant portion of the area is seen as very high risk according to the model. On the other hand, SDA predicts the smallest area for this category with 14.25%, highlighting its more restrained assessment of very high-risk zones. In XGB, the coverage in terms of “very high,” “high,” “moderate,” “low,” and “very low,” classes is 17.59% (183.72 km^2^), 14.33% (149.66 km^2^), 14.48% (151.23 km^2^), 18.24% (190.51 km^2^), and 35.36% (369.32 km^2^), respectively (Table 5). The areas covered by the “very low,” “low,” “moderate,” “high,” and “very high” classes were 36.48%, 18.17%, 15.09%, 14.13%, and 16.13%, respectively, for the GBM model. For the BAM model, the corresponding area was 33.55%, 17.95%, 17.19%, 15.66%, and 15.65%. With the MARS model, the area falls were 32.09%, 19.39%, 17.78%, 15.20%, and 15.54% (Fig. 6).

The comparison of Table 4 (resampling factor 5) and Table 5 (resampling factor 10) revealed that the choice of resampling factor significantly impacted the overall performance of the rFerns model for flood hazard zoning. The rFerns exhibited significantly higher standard deviations compared to other models for each flood hazard zone category, indicating an inconsistency in predictions for both resampling factors. The rFerns model assigned the largest area to the “very high” category of flood hazard class, covering approximately 32.78% (3423.26 km^2^), and the “very low” flood hazard class, covering about 2.94% (306.66 km^2^) (Fig. 5). In RF, the coverage in terms of “very low,” “low,” “moderate,” “high,” and “very high,” flood hazard classes is 34.54% (360.73 km^2^), 18.69% (195.20 km^2^), 16.85% (176.00 km^2^), 16.61% (173.44 km^2^), and 13.32% (139.08 km^2^), respectively. The AdaBoost ML estimated that 25.69% (268.33 km^2^) of the study area has a “very low” category for flood hazard zone. The “low,” and “moderate” hazard zones covered 20.87% (217.93 km^2^) and 20.02% (209.05 km^2^) of the area, respectively (Table 4), while the remaining 19.23% (200.85 km^2^) and 14.20% (148.28 km^2^) fall into the “high,” and “very high” flood hazard classes.

**Table 4:** Areal coverage by ML models (resampling 5) for flood hazard zoning

Model	Unit	Very low	Low	Moderate	High	Very high
RF	Sq km	360.73	195.20	176.00	173.44	139.08
	%	34.54	18.69	16.85	16.61	13.32
AdaBoost	Sq km	268.33	217.93	209.05	200.85	148.28
	%	25.69	20.87	20.02	19.23	14.20
rFerns	Sq km	306.66	313.64	6232.62	168.26	3423.26
	%	2.94	3.00	59.67	1.61	32.78
XGB	Sq km	341.54	191.89	174.65	162.36	174.01
	%	32.70	18.37	16.72	15.55	16.66
DeepBoost	Sq km	369.32	190.51	151.23	149.66	183.72
	%	35.36	18.24	14.48	14.33	17.59
GBM	Sq km	361.81	192.03	158.66	151.86	180.08
	%	34.64	18.39	15.19	14.54	17.24
SDA	Sq km	253.02	220.32	233.17	192.60	145.33
	%	24.23	21.09	22.32	18.44	13.91
BAM	Sq km	348.74	187.30	177.32	163.59	167.49
	%	33.39	17.93	16.98	15.66	16.04
monmlp	Sq km	402.09	151.07	129.45	122.13	239.70
	%	38.50	14.46	12.39	11.69	22.95
MARS	Sq km	336.15	205.60	180.36	159.37	162.96
	%	32.18	19.69	17.27	15.26	15.60
Average % of areal coverage	%	29.4	16.9	21.7	14.0	18.6
Std. dev of % areal coverage	%	10.3	5.3	13.8	4.9	5.9

**Table 5:** Areal coverage by ML models (resampling 10) for flood hazard zoning

Model	Unit	Very low	Low	Moderate	High	Very high
RF	Sq km	355.33	167.16	170.71	192.82	158.43
	%	34.02	16.00	16.34	18.46	15.17
AdaBoost	Sq km	261.33	227.51	204.62	200.11	150.88
	%	25.02	21.78	19.59	19.16	14.45
rFerns	Sq km	452.47	86.62	70.40	175.55	259.41
	%	43.32	8.29	6.74	16.81	24.84
XGB	Sq km	369.32	190.51	151.23	149.66	183.72
	%	35.36	18.24	14.48	14.33	17.59
DeepBoost	Sq km	352.35	168.56	168.39	192.67	162.47
	%	33.74	16.14	16.12	18.45	15.56
GBM	Sq km	381.05	189.76	157.61	147.56	168.46
	%	36.48	18.17	15.09	14.13	16.13
SDA	Sq km	252.61	219.87	233.40	189.76	148.80
	%	24.19	21.05	22.35	18.17	14.25
BAM	Sq km	350.45	187.51	179.49	163.56	163.43
	%	33.55	17.95	17.19	15.66	15.65
monmlp	Sq km	402.28	153.32	128.99	122.63	237.23
	%	38.52	14.68	12.35	11.74	22.71
MARS	Sq km	335.18	202.47	185.73	158.72	162.34
	%	32.09	19.39	17.78	15.20	15.54
Average % of areal coverage	%	33.6	17.2	15.8	16.2	17.2
Std. dev of % areal coverage	%	5.7	3.8	4.2	2.4	3.6

The flood hazard map created by the XGB model (Fig. 5) showed that 174.01 km^2^ (16.66%) of the study area has a “very high” category of flood hazard class. The “low” and “very low” flood hazard classes accounted for 32.70% and 18.37% of the study area, respectively. The “moderate” category of flood hazard class covered 174.65 km^2^ (16.72%) of the area. According to the DeepBoost model, the study area has 17.59% (183.72 km^2^) in the “very high” flood hazard zone, followed by 14.33% (149.66 km^2^) in the “high” flood hazard class, 14.48% (151.23 km^2^) in the “moderate” hazard zones, 18.24% (190.51 km^2^) in the “low,” and 369.32 km^2^ (35.36%) in the “very low” flood hazard classes, respectively (Table 4). The spatial results of flood hazard via combined GBM and SDA showed that 31.15% of the area had “very high” flood hazard zone (Fig. 5). Moreover, 37.51% had “moderate” hazard zones, whereas “low” and “very low” flood hazard classes were found to have 58.87% and 39.48% areas, respectively. The BAM ML model showed that “very low,” “low,” “moderate,” “high,” and “very high” category of flood hazard classes cover 33.39% (348.74 km^2^), 17.93% (187.30 km^2^), 16.98% (177.32 km^2^), 15.66% (163.59 km^2^), and 16.04% (167.49 km^2^) of the entire study area, respectively (Fig. 5). The areas occupied by the “very high,” “high,” “moderate,” “low,” and “very low,” classes were 22.95%, 11.69%, 12.39%, 14.46% and 38.50%, respectively, for the monmlp model. For the MARS model, the corresponding values were 15.60%, 15.26%, 17.27%, 19.69%, and 32.18% (Fig. 5).

Model monmlp exhibited more consistency between the resampling factors, followed by SDA, BAM, and MARS. These results indicated the areal coverage of different flood hazard zones in the study area and highlighted variations in susceptibility to flooding across different ML models.

For resampling factor 5, statistical analysis has uncovered meaningful insights into model performance across categories. The “high” category shows the lowest standard deviation at 4.9 followed by the “low” category, indicating a high degree of consistency among models and remarkable uniformity in this category. For resampling factor 10, the “high” category still exhibits the lowest standard deviation, which is 2.40. This reaffirms that the models performed most similarly in the “high” category. The “low” category also maintains a relatively low standard deviation of 3.80, indicating that the models performed fairly similarly in this category as well. Therefore, based on the standard deviations, the models performed most equally in the “high” category, followed by the “low” category.

The analysis of models’ estimations of areal coverage in percentage in two scenarios, namely Table 4 with a resampling factor of 5 and Table 5 with a resampling factor of 10, shed light on the variations in flood hazard predictions. In the “ very low” flood hazard category, there was a noticeable increase in the estimated coverage in Table 5, indicating that, on average, the models predicted a higher percentage of “very low” flood hazard areas with a higher resampling factor. Similarly, the “low” category showed a slight increase in estimated coverage in Table 4, suggesting that the models predicted a slightly higher percentage of low flood hazard areas.

However, the “moderate” flood hazard category stood out with a significant decrease in estimated coverage in Table 5. This reduction in the predicted percentage of moderate flood hazard areas was a notable change and highlighted the sensitivity of predictions to changes in the resampling factor. In contrast, the “high” flood hazard category demonstrated an increase in estimated coverage in Table 5, indicating a higher percentage of high flood hazard areas. Conversely, the “very high” category showed a decrease in estimated coverage in Table 4, suggesting a slightly lower percentage of “very high” flood hazard areas.

The comparison revealed that the choice of resampling factor substantially impacted model predictions, especially in the moderate flood hazard category. This underscored the need to carefully evaluate the resampling factor’s influence on predictions when applying machine learning models to flood hazard zoning. The study also identified Khanakul I and Khanakul II blocks (Figs. 1, 5, and 6) as the most vulnerable to flood-prone areas, particularly in the lower parts of the study area during the rainy season with bank failure, with a particular emphasis on the influence of major streams and their adjacent areas on the susceptibility to flooding. The different ML models made varying predictions of flood risk across the study area. Comparing their outputs allowed the assessment of uncertainty and variability in the flood hazard zoning maps. The analysis indicated that some models were more sensitive to resampling than others.

### Model validation analysis

Table 6 presents the performance metrics of various ML models for flood hazard zone classification, considering two different resampling factors (5 and 10). These metrics provide insights into the models' effectiveness in correctly classifying flood hazard zones. The accuracy of the models, which measures the proportion of correctly classified cases, varies among the models and resampling factors. RF at resampling 5 displays the best overall performance, with high accuracy, sensitivity, area under the AUC curve, and well-balanced specificity. It would serve as a robust candidate model when reducing false negative predictions is critical. PPV measures the proportion of positive cases correctly identified by the model, while NPV represents the proportion of negative cases correctly identified. These values are crucial for assessing the rates of false positives and false negatives. RF at resampling factor 5 exhibits a PPV of 0.741, signifying a relatively low rate of false positives. In contrast, AdaBoost maintains consistent PPV values at both resampling factors, indicating a similar rate of correctly identifying positive cases. NPV values also remain stable for AdaBoost, reflecting a stable rate of correctly identifying negative cases. AdaBoost exhibits comparable performance to RF, albeit with marginally lower sensitivity and higher specificity. Its low false positive rate makes it suitable if avoiding false alarms is prioritized.

**Table 6:** Confusion matrix of the employed ML models

Model	Resampling	Accuracy	Kappa	Sensitivity	Specificity	PPV	NPV	AUC
RF	10	0.753	0.492	0.866	0.617	0.731	0.794	0.812
	5	0.778	0.557	0.853	0.704	0.741	0.829	0.847
AdaBoost	10	0.770	0.540	0.824	0.717	0.742	0.805	0.839
	5	0.771	0.541	0.808	0.733	0.750	0.795	0.837
rFerns	10	0.747	0.495	0.806	0.690	0.720	0.782	0.839
	5	0.745	0.490	0.799	0.692	0.719	0.776	0.839
XGB	10	0.764	0.528	0.837	0.692	0.729	0.811	0.835
	5	0.761	0.522	0.830	0.693	0.728	0.805	0.822
DeepBoost	10	0.766	0.532	0.827	0.705	0.735	0.805	0.835
	5	0.762	0.524	0.829	0.695	0.729	0.804	0.835
GBM	10	0.759	0.518	0.823	0.695	0.728	0.799	0.827
	5	0.757	0.515	0.829	0.687	0.724	0.802	0.823
SDA	10	0.739	0.477	0.805	0.673	0.709	0.777	0.807
	5	0.739	0.478	0.805	0.674	0.710	0.777	0.807
BAM	10	0.753	0.506	0.814	0.693	0.724	0.790	0.816
	5	0.752	0.505	0.814	0.691	0.723	0.790	0.816
monmlp	10	0.745	0.490	0.830	0.661	0.708	0.797	0.809
	5	0.745	0.490	0.829	0.662	0.708	0.796	0.809
MARS	10	0.746	0.492	0.794	0.698	0.722	0.774	0.815
	5	0.748	0.497	0.799	0.698	0.724	0.778	0.815

The AUC represents a model’s ability to distinguish between positive and negative classes at different classification thresholds. It plots the true positive rate (sensitivity) against the false positive rate (1—specificity) for various threshold settings. The AUC quantifies the overall discriminative power of the model: a higher AUC value indicates a better ability of the model to differentiate between the two classes. RF at resampling factor 5 attains an AUC of 0.847, implying strong discriminative performance. Similarly, AdaBoost consistently demonstrates good discriminative ability, with AUC values of 0.837 at factor 5 and 0.839 at factor 10. A perfect model would have an AUC of 1, meaning it can separate positive and negative cases perfectly. An AUC of 0.5 suggests that the model’s performance is no better than random chance.

XGB follows random forest and AdaBoost in terms of accuracy, with higher sensitivity but lower specificity than AdaBoost. Its decent positive predictive value indicates reliable positive predictions, making it a reasonably balanced model. DeepBoost is similar to XGB, with slightly lower accuracy but improved specificity.

Lastly, the SDA model consistently lags behind most other models across a range of evaluation metrics. It can be concluded that ensemble methods, especially RF, AdaBoost, and XGB, deliver optimal performance for the classification task, with the choice among them dependent on sensitivity, specificity, or predictive value deemed more integral based on the problem context.

### Parameter sensitivity analysis

**Fig. 7:**
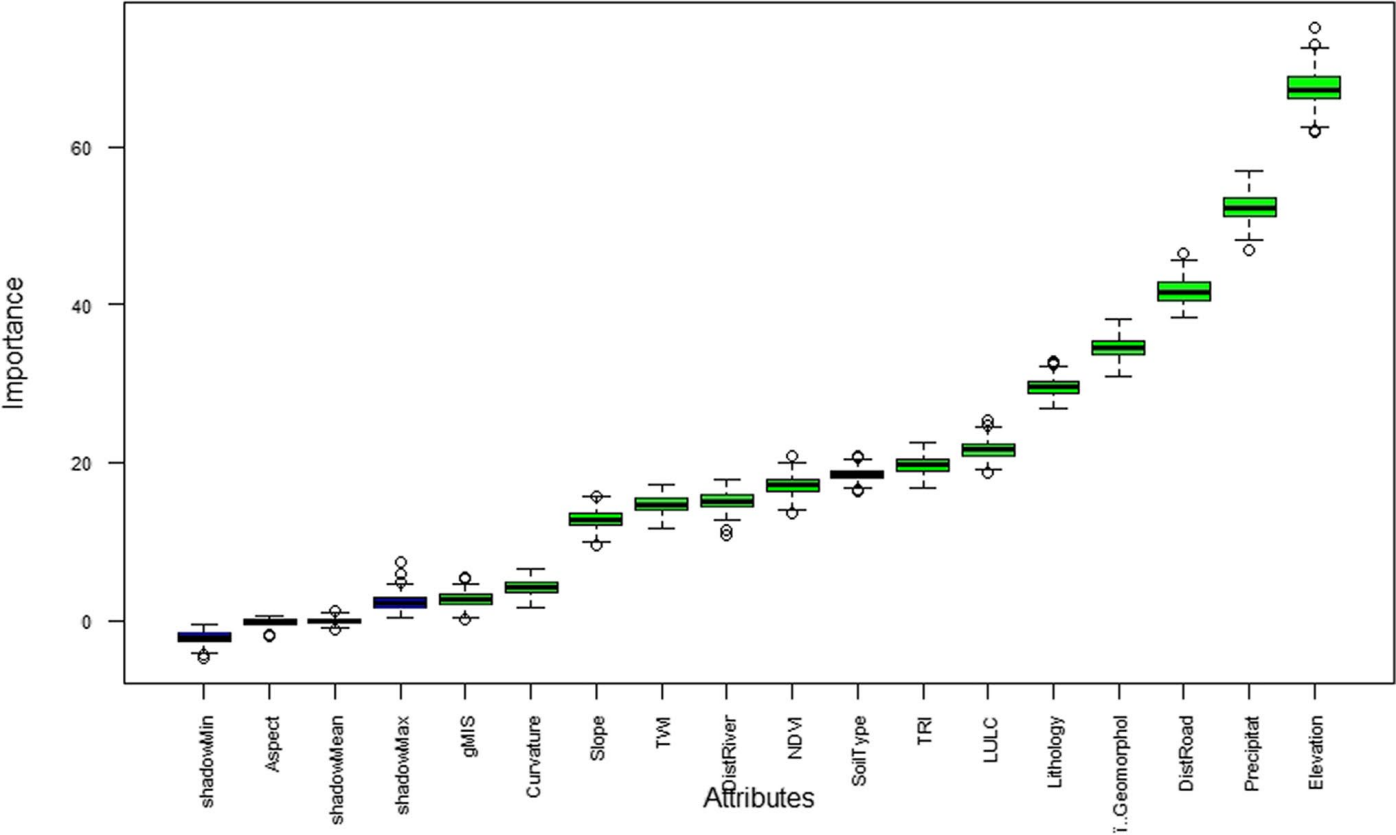
Boruta analysis for flood hazard prediction

In Table 7, the mean importance column shows the average importance measure (IMp) calculated across multiple iterations. The Median Importance column displays the median IMp. The Min Importance column indicates the minimum IMp, while the Max Importance column reveals the maximum IMp. The Norm Hits column represents the number of times a feature was deemed more important than the shadow feature, normalized by the total importance counts. This involves creating duplicates of the original attributes by randomly mixing the features, known as Shadow Attributes. Boruta performed 500 iterations and stopped in 23.88438 min. Here, 14 attributes were confirmed as important factors and one attribute was confirmed as unimportant (Fig. 7). Boruta performed 500 iterations by the RF algorithm. Based on the Boruta parameter sensitivity analysis, the maximum mean importance was found for elevation (67.41) followed by precipitation (52.35), distance to road (41.67), geomorphology (34.43), lithology (29.53), LULC (21.68), TRI (19.76), soil type (18.54), NDVI (17.17), distance to river (15.09), TWI (14.68), slope (12.73), curvature (4.10), and gMIS (2.64) among the flood conditioning factors (Table 7). In this study, all fourteen features considered were found to be more important than the shadow feature, where the aspect factor was deemed less important in determining flood hazard mapping. The median importance of the among factors varies from -0.23 (aspect) to 67.25 (elevation). The maximum important (maxImp) factor selected by Boruta is elevation (75.02), followed by precipitation (56.83), distance to road (46.56), geomorphology (38.07), lithology (32.81), LULC (25.33), TRI (22.53), NDVI (20.9), soil type (20.87), distance to river (17.79), TWI (17.26), slope (15.73), curvature (6.6), gMIS (5.54), and aspect (0.64) respectively. However, the minImp value ranged from 61.89 (elevation) to − 2.06 (aspect). No significant importance was found in the flood hazard model’s depiction of the aspect factor (− 0.34). This analysis reveals that elevation was the most significant factor in assessing flood hazard probability. The study area demonstrated that the upstream region, being at a higher elevation, had a lower flood risk, whereas the downstream region, characterized by flat terrain, was more vulnerable to flood hazards.

**Table 7:** Boruta feature importance analysis

Factor	meanImp	medianImp	minImp	maxImp	normHits	decision
Geomorphology	34.43	34.42	30.87	38.07	1.00	Confirmed
gMIS	2.64	2.67	0.19	5.54	0.59	Confirmed
Lithology	29.53	29.55	26.90	32.81	1.00	Confirmed
LULC	21.68	21.60	18.60	25.33	1.00	Confirmed
NDVI	17.17	17.11	13.50	20.90	1.00	Confirmed
Precipitation	52.35	52.24	46.95	56.83	1.00	Confirmed
Slope	12.73	12.75	9.46	15.73	1.00	Confirmed
SoilType	18.54	18.51	16.33	20.87	1.00	Confirmed
TRI	19.76	19.78	16.83	22.53	1.00	Confirmed
TWI	14.68	14.60	11.74	17.26	1.00	Confirmed
Aspect	-0.34	-0.23	-2.06	0.64	0.00	Rejected
Elevation	67.41	67.25	61.89	75.02	1.00	Confirmed
Curvature	4.10	4.09	1.65	6.60	0.93	Confirmed
DistRiver	15.09	15.06	10.71	17.79	1.00	Confirmed
DistRoad	41.67	41.64	38.35	46.56	1.00	Confirmed

**Fig. 8:**
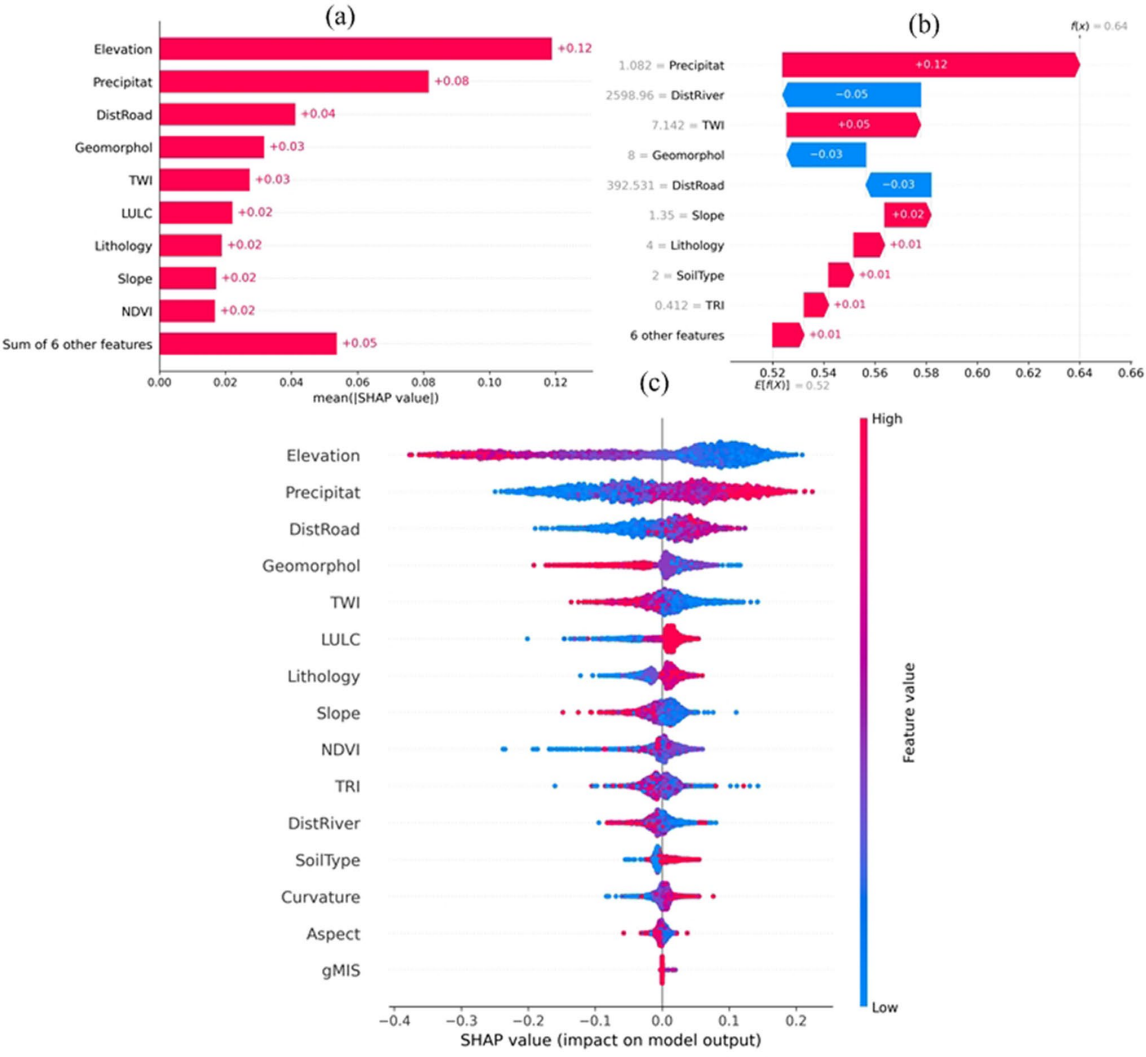
SHAP analysis for flood hazard prediction

According to the SHAP analysis, the local explainability is an XAI (explainable AI) technique that allows an analyst to identify the factors contributing to a given prediction. It can also analyze the factors that affect a single prediction. Based on RF-based SHAP bar plots, elevation (0.12), precipitation (0.08), distance to road (0.04), geomorphology (0.03), and TWI (0.03) are the top five most important factors for the flood occurrences (Fig. 8a). Furthermore, LULC, lithology, slope, and NDVI exhibit moderate impacts on flood hazard probabilities, whereas distance to river, aspect, curvature, soil type, TRI, and gMIS factor are relatively less influential in the decision-making process for flood hazard assessment.

Although elevation and rainfall have a major and significant positive impact on flood hazard analysis from the SHAP score, the distance to the road also plays a crucial role. Areas further from roads are shown by models to have a lower likelihood of flooding. The NDVI, slope, lithology, and LULC components seem to contribute less favorably to flood hazard assessment. Using the SHAP waterfall plots, the color pink represents positive SHAP values, while blue indicates negative SHAP values, illustrating their impact on the model output. The SHAP waterfall plot revealed that the precipitation (0.12), TWI (0.05), and slope (0.02) are the most positive effective factors to flooding while the distance to river (− 0.05), geomorphology (− 0.03), and the distance to road (− 0.03) are negative impacts on flood occurrence (Fig. 8b). Moreover, lithology, soil type, land use/land cover (LULC), topographic roughness index (TRI), normalized difference vegetation index (NDVI), aspect, curvature, and gMIS have a marginal impact, with values close to or less than + 0.02, on the flood hazard assessment. Through the SHAP summary plots, the blue indicated the low SHAP value, and the pink indicated the high SHAP value for impact on model output. The mean absolute SHAP value was computed to assess each feature’s impact on the model’s predictions, aiming to elucidate their relative importance in decision-making. The parameters are arranged in descending order, prioritizing those with a more pronounced influence on the final prediction. Figure 8c displays SHAP values for input factors, demonstrating their impact trends. On the x-axis are the SHAP values, while the y-axis represents the conditioning factors. Each sample is depicted as a dot, where blue denotes lower values, and pink denotes higher ones. The horizontal position indicates whether the conditioning factor positively or negatively affects the prediction. The values of pink-colored precipitation and elevation can also influence a flood hazard model’s ability to predict (Fig. 8c). The findings reveal that as elevation decreases and precipitation increases, the extent of water area and inundation surface generally increases, leading to a higher probability of flooding. Conversely, higher values for DistRiver, DistRoad, TRI, NDVI, slope, LULC, and curvature are linked to a reduced likelihood of flooding. Moreover, gMIS and Aspect contribute the least to the model’s predictions for flood hazard assessment. Similarly, soil type and TWI positively affect flood potential, as areas with better soil permeability distribution have greater chances of agricultural productivity. However, the TRI, NDVI, soil type, lithology, slope, curvature, gMIS, and aspect component appear to have a minimal favorable impact on the models. Still, their influence does not seem important in the flood hazard mapping.

### Impact of the flood

**Fig. 9:**
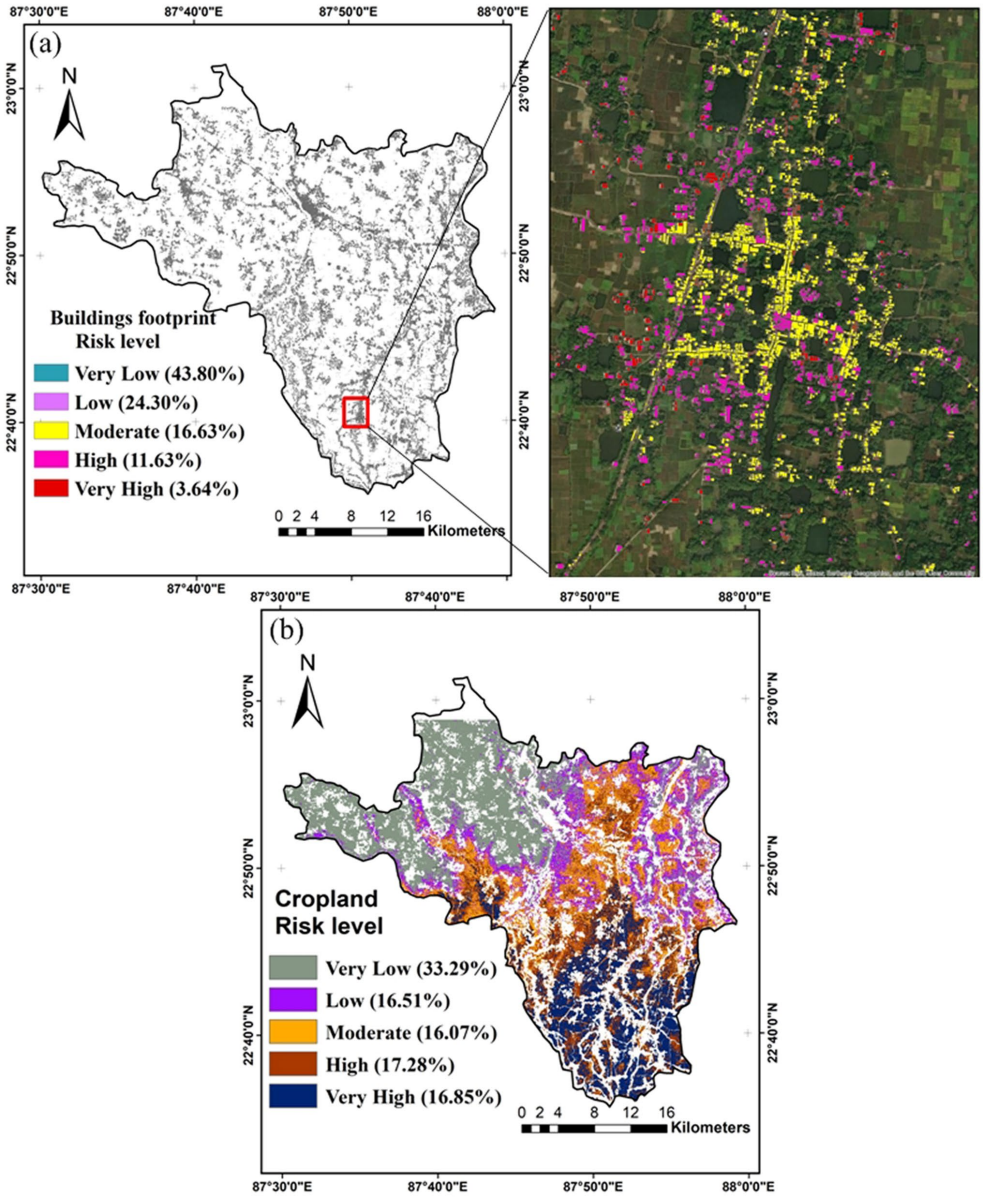
Impact of flood hazard **a** risk of building footprint and **b** cropland risk

After achieving the revelation of flood disturbance of building footprint from the Open Buildings V3 Polygons dataset (URL: https://sites.research.google/open-buildings/), the probability of building damage extent could be resultant for each building over the entire area. In this study, a total 449, 194 of building footprints open dataset were acquired from images of a high resolution of 50 cm with a > 0.65 confidence score. Figure 9a shows the Rajhati (Khanakul II) for flood hazard impacts on the region’s humanitarian response and properties. Accordingly, the whole region is subjected to flood hazards and building vulnerability, ranging from areas with very high to very low risks. However, the majority of the building footprints (i.e., 15.27%) are subjected to high- and very-risk probability, followed by buildings with very low (i.e., 43.80%) and low (i.e., 24.30%) and moderate risk (i.e., 16.63%), respectively (Fig. 9a). Similarly, the cropland area affected by the flood in this region could be categorized into five risk classes namely very high (16.85%), high (17.28%), moderate (16.07%), low (16.51%), and very low (33.29%) respectively (Fig. 9b). This study provides a comprehensive understanding of the potential impacts and existences of flood hazard. It also helps in developing effective strategies and managing the risks associated with floods, like buildings and crops. It is important that the various authorities, developers, and urban planners are aware of the risks associated with flood hazards.

## Conclusion

The presented research has successfully conducted an extensive flood hazard assessment for the Arambag region by integrating advanced machine learning techniques and multi-sourced spatial data. Leveraging Sentinel-1 SAR imagery, the Global Flood Database, and in situ surveys, a robust flood inventory was constructed to characterize both long-term and event-specific inundation patterns. This holistic approach comprehensively represented the study area’s flood characteristics. The study meticulously incorporated 15 flood conditioning factors encompassing terrain attributes, soil composition, land cover, climatic variables, and anthropogenic factors to account for all potential influential parameters. Rigorous validation was undertaken using various statistical performance metrics, including accuracy, AUC, kappa, sensitivity, specificity, and cross-validation techniques, adding credence to the results. State-of-the-art machine learning algorithms, including RF, AdaBoost, rFerns, XGB, DeepBoost, GBM, SDA, BAM, monmlp, and MARS, were explored given their enhanced modeling capabilities over conventional approaches. Random Forest was identified as the optimal technique for delineating the complex flood dynamics of the region. To eliminate redundancies, model refinement was achieved through input variable selection techniques, including OLS regression, multicollinearity analysis, and nature-inspired algorithms. The OLS regression determined precipitation, geomorphology, elevation, lithology, and TWI as the most significant factors. The multicollinearity test using VIF and TOL ensured no redundancy among input factors. Furthermore, optimization algorithms like PSO, GA, GSO, HHO, and GWO were implemented, which provided an ideal subset of factors for modeling. The Boruta and SHAP methods were finally used to evaluate variable importance and sensitivity. The study revealed varying levels of flood hazard impact on building footprints and cropland in the region. Most building footprints faced high to very high flood risk, with varying degrees of vulnerability. Similarly, cropland areas exhibited different risk levels, with a significant portion in the very low-risk category.

This research has proposed a sophisticated framework for flood hazard modeling by leveraging cutting-edge data resources and algorithms. The generated flood maps will be valuable for risk-informed planning and mitigation in Arambag. This study significantly contributes to flood hazard mapping research through the synergistic integration of diverse data, implementation of advanced models, and predictive variable analysis. The future scope of the study employed more data-driven datasets with CMIP6-based climate change analysis based advanced hybrid deep learning, internet of things (IoT), and unmanned aerial system (UAS) approach.

### Supplementary Information

Below is the link to the electronic supplementary material.

Supplementary file1 (DOCX 200 KB)

## Data Availability

The data supporting this study’s findings are available from the corresponding author, [Quoc Bao Pham, quoc_bao.pham@us.edu.pl], upon reasonable request.
