# Supplementary material for: Integrating machine learning and geospatial data analysis for comprehensive flood hazard assessment
Source: Environ Sci Pollut Res Int. 2024 Jul 20;31(35):48497–522. doi: 10.1007/s11356-024-34286-7 (PMC11297827; doi:10.1007/s11356-024-34286-7)
Supplement: Supplementary file 1 — Supplementary file1 (DOCX 200 KB) [file 11356_2024_34286_MOESM1_ESM.docx]

**Supplementary Materials:**

Supplementary Table.1 Best feature selection and hyper-parameters tunned of the light gbm optimization based metaheuristic algorithm for flood hazard susceptibility mapping

| Algorithm name | Best features | Tuning parameters |
| --- | --- | --- |
| PSO | 'Geomorphology',  'Elevation',  'Lithology',  'TRI',  'TWI',  'Precipitation',  'Slope',  'SoilType',  'Curvature',  'NDVI',  'DistRoad',  'DistRiver',  'gMIS' | n_iteration=20, Total Bins 1752, pavg=0.498174 -> initscore=-0.007303, population_size=20, Finished iteration #19 with objective value 0.30708779035092854. Current best value is 0.30708779035092854 |
|  |  |  |
| GSO | 'Geomorphology',  'Elevation',  'TRI',  'Precipitation',  'SoilType',  'NDVI',  'LULC',  'Aspect',  'DistRoad',  'DistRiver' | n_iteration=50, population_size=50, g0=100, eps=0.5, minimize=True, Total Bins 520, Number of data points in the train set: 6847, number of used features: 3, pavg=0.498174 -> initscore=-0.007303, Start training from score -0.007303,Finished iteration #9 with objective value 0.39013012872392006. Current best value is 0.3251658438571509 |
|  |  |  |
|  |  |  |
|  |  |  |
|  |  |  |
|  |  |  |
| GA | 'Geomorphology',  'Elevation',  'Lithology',  'TRI',  'TWI',  'Precipitation',  'Slope',  'SoilType',  'Curvature',  'NDVI',  'LULC',  'Aspect',  'DistRoad',  'DistRiver',  'gMIS' | n_iteration=20,  population_size=20,selective_pressure=2,elitism=4, mutation_rate=0.1,minimize=True  -05, Total Bins 1873, Number of data points in the train set: 6847, number of used features: 13, pavg=0.498174 -> initscore=-0.007303,Finished iteration #19 with objective value 0.30715234360321764. Current best value is 0.30715234360321764 |
|  |  |  |
|  |  |  |
|  |  |  |
|  |  |  |
|  |  |  |
|  |  |  |
|  |  |  |
|  |  |  |
| HHO | 'Geomorphology',  'Elevation',  'Lithology',  'TRI',  'TWI',  'Precipitation',  'Slope',  'SoilType',  'Curvature',  'NDVI',  'DistRoad',  'DistRiver',  'gMIS' | n_iteration=20,population_size=20,minimize=True  Finished iteration #19 with objective value 0.30715234360321764. Current best value is 0.30708779035092854 |
|  |  |  |
|  |  |  |
| GWO | 'Geomorphology',  'Elevation',  'Lithology',  'TRI',  'TWI',  'Precipitation',  'Slope',  'SoilType',  'Curvature',  'NDVI',  'LULC',  'Aspect',  'DistRoad',  'DistRiver' | n_iteration=20,population_size=20,minimize=True  Total Bins 2095, Number of data points in the train set: 6847, number of used features: 14, pavg=0.498174 -> initscore=-0.007303,Start training from score -0.007303 Finished iteration #19 with objective value 0.30715234360321764. Current best value is 0.30715234360321764 |

| 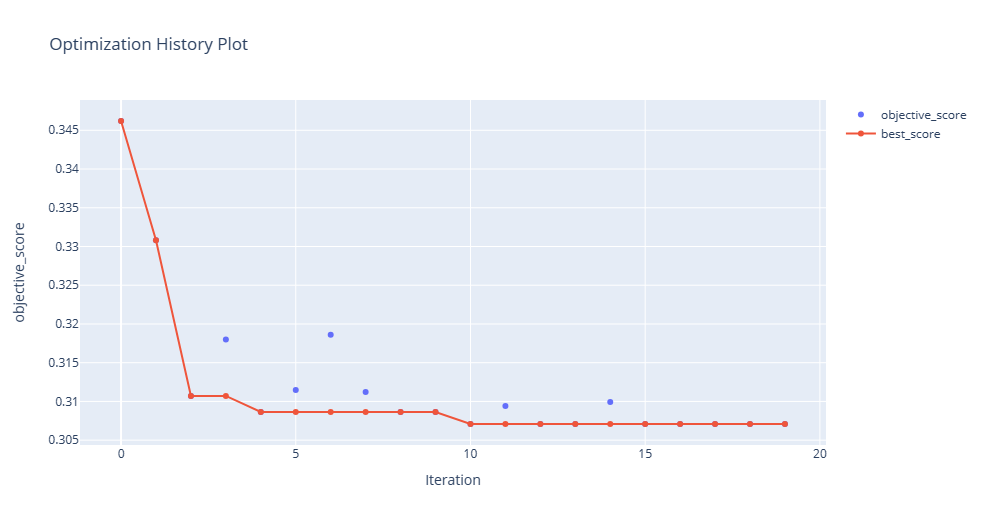  (a) |
| --- |
| 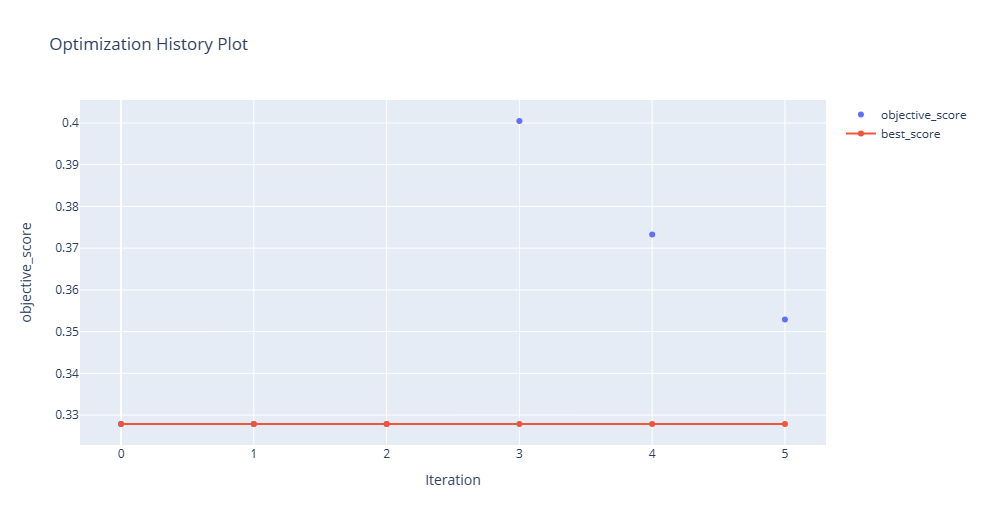  (b) |
| 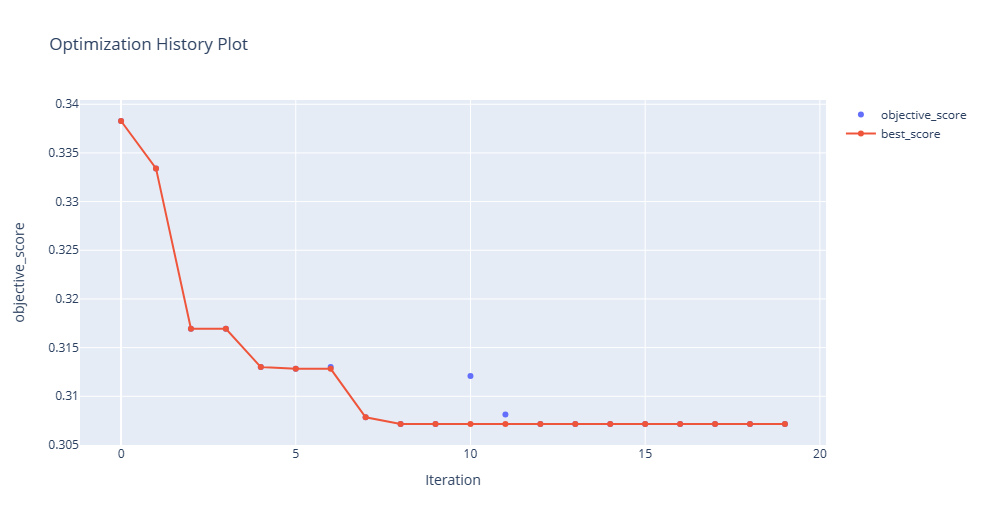  (c) |
| 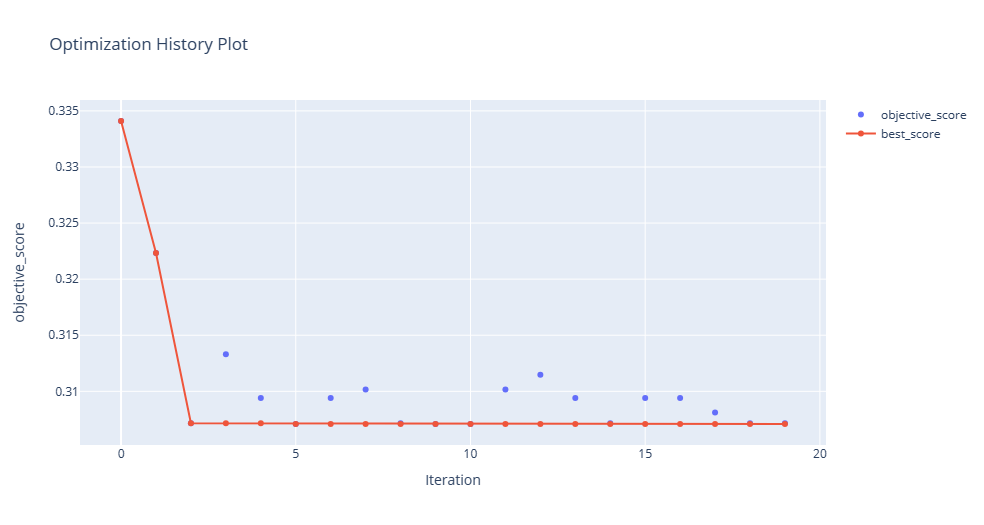  (d) |
| 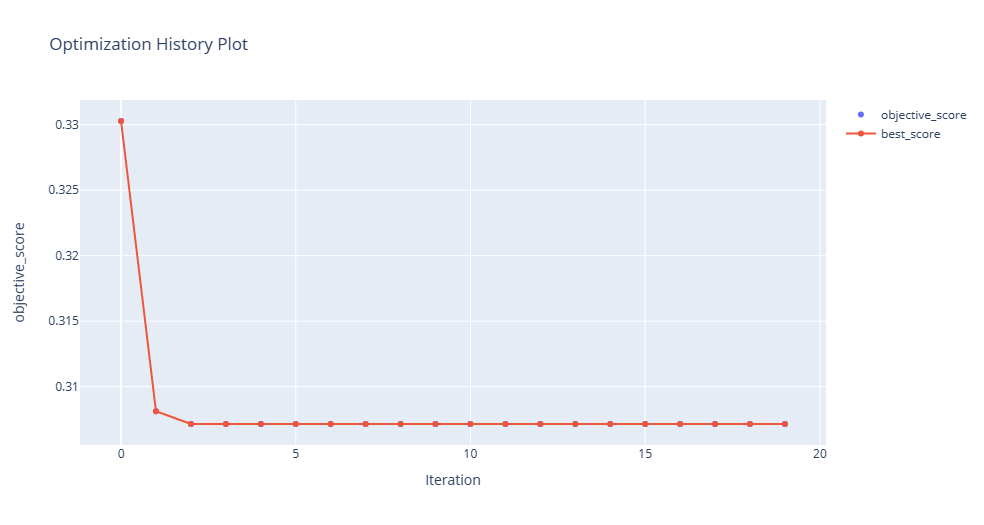  (e) |

Supplementary Figure.1 Optimization history plot (a) PSO (b) GSA (c) GA (d) HH and (e) GWO
